# Identification of drought responsive *Elaeis guineensis WRKY* transcription factors with sensitivity to other abiotic stresses and hormone treatments

**DOI:** 10.1186/s12864-022-08378-y

**Published:** 2022-02-26

**Authors:** Fong Chin Lee, Wan Chin Yeap, David Ross Appleton, Chai-Ling Ho, Harikrishna Kulaveerasingam

**Affiliations:** 1 Sime Darby Plantation Technology Centre Sdn. Bhd, 43400 Serdang, Selangor Malaysia; 2 Department of Cell and Molecular Biology, Faculty of Biotechnology and Biomolecular Sciences, Universiti Putra Malaysia (UPM), 43400 Serdang, Selangor Malaysia; 3 Sime Darby Research Sdn Bhd, R&D Centre, 42700 Banting, Selangor Malaysia

**Keywords:** Oil palm, Drought, Salinity, Heat, Abscisic acid, Salicylic acid, Hydrogen peroxide, Group III WRKY, Reactive oxygen species

## Abstract

**Background:**

The ability of plants to withstand and thrive in an adverse environment is crucial to ensure their survivability and yield performance. The WRKY transcription factors (TFs) have crucial roles in plant growth, development and stress response, particularly drought stress. In oil palm, drought is recognized as one of the major yield limiting factors. However, the roles of WRKY TFs in the drought response of oil palm is unclear.

**Results:**

Herein, we studied the transcriptome of drought treated oil palm leaf and identified 40 differentially expressed genes (DEGs) of *WRKY* TFs, of which 32 DEGs were upregulated and 8 DEGs were downregulated in response to drought stress in oil palm. They were categorized into Groups I to IV based on the numbers of WRKY domain and the structural difference in the zinc finger domain. Multiple stress- and hormone-responsive *cis*-regulatory elements were detected in the drought responsive oil palm *EgWRKY* (Dro-*EgWRKY*) genes. Fourteen of the 15 selected oil palm *WRKY* (*EgWRKY*) genes demonstrated a tissue-specific expression profile except for *EgWRKY28* (Group I), which was expressed in all tissues tested. The expression levels of 15 candidate *EgWRKYs* were upregulated upon salinity and heat treatments, while several genes were also inducible by abscisic acid, methyl jasmonate, salicylic acid and hydrogen peroxide treatments. Members of the Group III WRKY TFs including *EgWRKY07, 26, 40, 52, 59, 73* and *81* displayed multiple roles in drought- and salinity-response under the modulation of phytohormones.

**Conclusions:**

*EgWRKY* TFs of oil palm are involved in phytohormones and abiotic stress responses including drought, salinity and heat. *EgWRKY07*, *26*, *59* and *81* from Group III maybe important regulators in modulating responses of different abiotic stresses. Further functional analysis is required to understand the underlying mechanism of WRKY TFs in the regulatory network of drought stress.

**Supplementary Information:**

The online version contains supplementary material available at 10.1186/s12864-022-08378-y.

## Background

WRKY protein is one of the largest transcription factor (TF) family found in the plant kingdom. There are 197 WRKY members in *Glycine max* [1], 160 members in *Triticum aestivum* [2] and 145 members in *Brassica rapa* [3]. WRKY TF is characterized by the presence of a highly conserved WRKY domain comprising of two parts, the DNA-binding heptapeptide WRKYGQK and the zinc finger binding motif which spans about 60 amino acids in length [4]. Other forms of heptapeptide found in the WRKY TF include WRKYGKK, WKKYGQK, WRKYGQR and WRKYGEK [5]. The zinc finger structure can be divided based on the C2H2 motif (C-X4-5-C-X22-23-H-X1-H) and the C2HC motif (C-X5-7-C-X2-3-H-X1-C) [4]. The C-terminus of WRKY domain has been shown to have a high binding affinity to its cognate *cis*-acting element, designated as W-box (C/T)TGAC(T/C) via positively charged β-strands [4]. As a result, the WRKY proteins are categorized into three groups based on the number of WRKY domains and the zinc finger binding motifs. Group I members have two WRKY domains at both termini whereas Group II and III members have only one WRKY domain. Group I and II members share the same C2H2-type zinc finger motif while Group III members have the C2HC-type. Furthermore, Group II members can be divided into five sub-groups based on their phylogenetic relationships [6].

Being a TF superfamily, WRKY is involved in many biological processes at different stages of the plant life cycle with great emphasis on plant defence response towards biotic and abiotic stresses through the transcription regulation of stress-responsive genes modulated by phytohormones. WRKY TFs are functionally expressed during pollen development [7, 8], adventitious root formation [9], flowering [10], leaf senescence [11], callus development [12] and homeostasis of phosphate [13]. Numerous studies have reported the involvement of *WRKY*s in the response of plants to multiple abiotic stresses such as drought, submergence, heat, cold, salinity and ion toxicity in various plants [14–17], under the influence of phytohormone signals, particularly ABA. In *Arabidopsis*, *AtWRKY46* was upregulated by drought, salinity, SA and hydrogen peroxide (H_2_O_2_) treatments [18]. *GhWRKY41* [16] from *Gossypium hirsutum* responded positively to drought and salt stresses in transgenic *Nicotiana benthamiana via* modulation of reactive oxygen species [19] production in ABA-dependent manner. Extensive studies in *T. aestivum* disclosed multiple *WRKY*s involved in different abiotic stresses particularly in drought and salt stresses such as *TaWRKY1*, *TaWRKY33* [17] and *TaWRKY46* [20]. *TaWRKY46* exhibited an enhanced tolerance to mannitol treatment in transgenic *Arabidopsis* by increasing the expression of several stress-related genes, namely *Δ-1-pyrroline-5-carboxylate synthetase 1* (*P5CS1*), *dehydration-responsive 29B* (*RD29B*), *dehydration-response element-binding protein 2A* (*DREB2A*), *ABA-response element binding factor 3* (*ABF3*), *C repeat/dehydration-responsive element-binding factor 2 and 3* (*CBF2*, *3*), via ABA-dependent and ABA-independent pathways [20]. In *Fragaria vesca*, the expression of *FvWRKY42* was induced by salt, drought, SA, MeJA and ABA treatments, and overexpression of *FvWRKY42* enhanced salt and drought stress tolerance [21].

Oil palm is a highly productive oil crop contributing to approximately 40% of global vegetable oil demand as food, animal feed and fuel, produced from less than 5.0-5.5% of the total global oil crop area (approximately 425 Mha) in year 2020 [22]. Oil palm yield is critically affected by environmental factors, particularly drought stress resulting from low rainfall and extended dry seasons caused by climate change, such as the *El Niño* events. The severe El Niño events in 1997-1998 and 2015-2016 caused declination of palm oil yield [23]. Drought stress caused long term impacts including abnormal frond development, low floral sex ratio leading to yield loss [24], hence, it is imperative to understand the underlying molecular events that cause these responses during drought stress to improve oil palm adaptability and tolerance. Xiao et al. [25] reported 95 members of WRKY TFs in oil palm genome and 17 *EgWRKYs* upregulated at 2-fold or higher by cold stress based on the transcriptomic data [25]. Out of 17 *EgWRKYs* genes, six of these cold-responsive *EgWRKYs* were also found to be induced by drought and salinity stresses [25]. However, little is known about the repertoire of *WRKY* transcripts in the transcriptome of drought treated oil palms and the response of these *WRKY* genes to different phytohormones. We analysed the transcriptomes of oil palm seedlings under drought stress to identify differentially expressed *EgWRKY* TFs involved in drought stress response. Phylogenetics analysis and gene expression characterization of these genes in response abiotic stresses and phytohormones were also conducted to close the current knowledge gap as well as to evaluate the potential of oil palm WRKY TFs in conferring drought tolerance.

## Results

### Identification of Dro-*EgWRKY* genes from the transcriptome of drought-treated oil palms

**Fig. 1:**
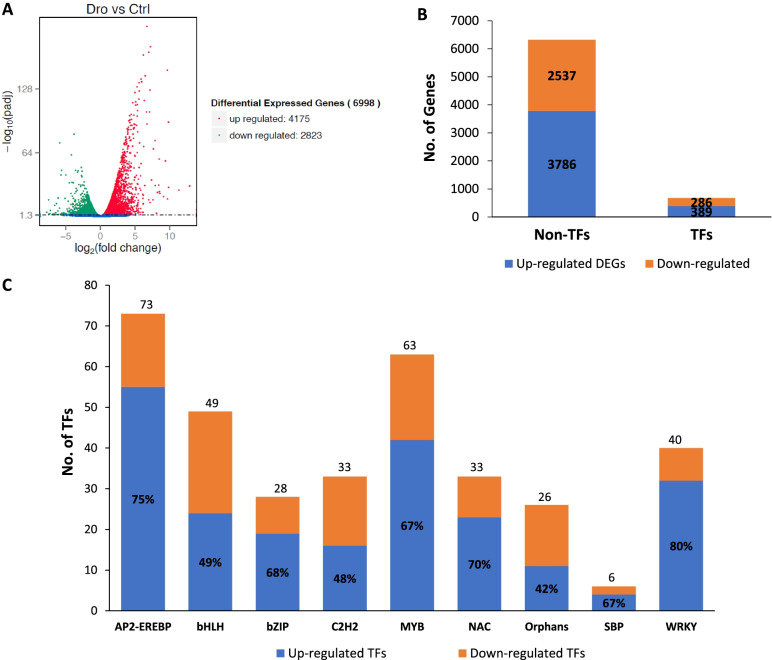
Differential expressed genes in response to drought stress. **A** Volcano plot of differentially expressed genes in drought (dro) vs control (ctrl). The x-axis shows the fold change in gene expression between different samples, and the y-axis shows the statistical significance of the differences. Among 6998 DEGs, 4175 DEGs were up-regulated and 2823 DEGs were down-regulated. Significantly up and down regulated genes are highlighted in red and green, respectively. Genes did not express differently between treatment group and control group are in blue. **B** Transcription analysis revealed approximately 10% of total up-regulated (389 TFs) and down-regulated DEGs (286 TFs) were of transcription factors from different TF families. **C** Further classification of drought response TFs into different transcription factor families. Significantly up-regulated and down-regulated of TFs and non-TFs are highlighted in blue and orange, respectively. Percentage represents total of up-regulated DEGs in the respective TF families

To elucidate the roles of TFs in drought response, we first identified the TFs among the DEGs identified from the RNA-Seq study on oil palm treated with drought stress in comparison to untreated control oil palm. A total of 6998 DEGs were identified from the RNA-seq analysis; whereby 4175 DEGs were upregulated and 2823 DEGs were downregulated in response to drought stress (Fig. 1A). Approximately 10% or 675 DEGs among the identified DEGs were TF genes; 389 were upregulated and 286 were downregulated in response to drought stress (Fig. 1B). They were categorized into different TF families, such as AP2-EREBP, bHLH, bZIP, C2H2, MYB, NAC, Orphans, SBP and WRKY which are known as stress-related TF families (Fig. 1C). Among the 40 *WRKYs* identified, 32 *WRKYs* were upregulated by drought stress, representing the highest percentage (80%) of TF family that was upregulated by drought stress while 8 *WRKYs* were downregulated (Fig. 1C). WRKY TFs have gained much attention recently in stress response particularly drought stress in many plants [26, 27]. Drought affects oil palm yield, however, limited knowledge is available on the function of *WRKYs* in drought stress response in oil palm. Thus, 40 differentially expressed *WRKY* genes were further analysed to understand their roles in abiotic stress response especially drought stress.

### Dro-*EgWRKY* are structurally classified into Group I-IV

The 40 drought responsive *EgWRKY* genes were named and categorized (Table 1) with reference to the oil palm *WRKY* TFs mentioned in [25]. The chromosomal locations of 3 *EgWRKY* genes (*EgWRKY84, 88* and *94*) which were not mapped earlier by Xiao et al. [25] were now successfully mapped to chromosome (CHR) 1, 2 and 15 respectively using the improved version of oil palm genome assembly [19]. *EgWRKY06* has the shortest open reading frame of 528 bp encoding for 176 amino acids while *EgWRKY18* has the longest open reading frame of 2214 bp encoding 738 amino acids. Most of the *EgWRKY* genes were predicted to be localized in the nucleus except for *EgWRKY56* which was predicted to be localized in the chloroplast. To gain insights into the functions of targeted *EgWRKY* genes, we searched for the rice WRKY proteins with the highest identities. Oil palm *EgWRKY* genes that have the highest sequence identities to the same rice *WRKY* gene may display a similar expression pattern in response to drought stress. For instance, *EgWRKY19, 63, 65*, *69* (that share the highest identities with *OsWRKY51*), *EgWRKY07, 26, 52*, *81* (that share the highest identities with *OsWRKY69*)*, EgWRKY34, 72, 80* (that share the highest identities with *OsWRKY24*) and *EgWRKY39, 47* and *84* (that share the highest identities with *OsWRKY29* gene), showed upregulation by drought stress (Table 1).

**Table 1:** List of 40 drought responsive *EgWRKY* genes in *E. guineensis* from leaf transcriptome

Grp	Gene name	Gene Locus ID	Log2Fold Change	p-value	CHR	ORF, bp	Size, aa	Intron	Exon	Conserved heptapeptide	Domain pattern	Zinc-finger type	Sub cellular localization	Orthologous gene	Locus name
I	*EgWRKY18*	LOC105038993	0.59173	0.0066	2	2214	738	4	5	2WRKYGQK	C-X_4-5_-C-X_22-23_-H-X_1_-H	C2H2	Nucleus	*OsWRKY30*	LOC_Os08g38990.3
I	*EgWRKY34*	LOC105044758	1.5654	8.6E-18	5	1782	594	4	5				Nucleus	*OsWRKY24*	LOC_Os01g61080.1
I	*EgWRKY43*	LOC105045782	1.1275	6.25E-09	5	1407	469	4	5				Nucleus	*OsWRKY87*	LOC_Os07g39480.1
I	*EgWRKY72*	LOC105052255	4.145	1.04E-17	10	1590	530	4	5				Nucleus	*OsWRKY24*	LOC_Os01g61080.1
I	*EgWRKY80*	LOC105057172	1.522	1.69E-13	14	1788	596	4	5				Nucleus	*OsWRKY24*	LOC_Os01g61080.1
I	*EgWRKY94*	LOC105034934	-0.684	0.0035	15	1923	641	5	6				Nucleus	*OsWRKY87*	LOC_Os07g39480.1
IIa	*EgWRKY66*	LOC105050724	-2.391	0.0412	8	771	257	3	4	1WRKYGQK	C-X_4-5_-C-X_22-23_-H-X_1_-H	C2H2	Nucleus	*OsWRKY76*	LOC_Os09g25060.1
IIa	*EgWRKY70*	LOC105051963	3.593	6.90E-20	9	876	292	4	5				Nucleus	*OsWRKY71*	LOC_Os02g08440.1
IIa	*EgWRKY88*	LOC105060191	1.269	0.0036	2	948	316	4	5				Nucleus	*OsWRKY76*	LOC_Os09g25060.1
IIb	*EgWRKY28*	LOC105041017	3.136	3.13E-60	3	1701	567	5	6				Nucleus	*OsWRKY01*	LOC_Os01g14440.1
IIb	*EgWRKY61*	LOC105048552	0.979	4.10E-05	7	1752	584	5	6				Nucleus	*OsWRKY01*	LOC_Os01g14440.1
IIc	*EgWRKY03*	LOC105055190	2.970	9.72E-08	1	588	196	2	3	1WRKYGQK/ 1WRKYGKK	C-X_4-5_-C-X_22-23_-H-X_1_-H	C2H2	Nucleus	*OsWRKY07*	LOC_Os05g46020.1
IIc	*EgWRKY06*	LOC105056374	-1.413	0.0282	1	528	176	2	3				Nucleus	*OsWRKY07*	LOC_Os05g46020.1
IIc	*EgWRKY22*	LOC105040295	0.814	0.0234	3	720	240	2	3				Nucleus	*OsWRKY102*	LOC_Os01g08710.2
IIc	*EgWRKY39*	LOC105044992	0.583	0.0072	5	933	311	2	3				Nucleus	*OsWRKY29*	LOC_Os07g02060.1
IIc	*EgWRKY44*	LOC105045639	-1.142	0.0066	5	942	314	2	3				Nucleus	*OsWRKY17*	LOC_Os01g74140.1
IIc	*EgWRKY47*	LOC105046637	1.208	0.0035	6	906	302	2	3				Nucleus	*OsWRKY29*	LOC_Os07g02060.1
IIc	*EgWRKY49*	LOC105047232	0.970	0.0363	6	1008	336	2	3				Nucleus	*OsWRKY11*	LOC_Os01g43650.1
IIc	*EgWRKY53*	LOC105047700	1.453	0.0005	6	606	202	2	3				Nucleus	*OsWRKY07*	LOC_Os05g46020.1
IIc	*EgWRKY56*	LOC105047934	1.203	0.0139	7	630	210	2	3				Chloroplast	*OsWRKY67*	LOC_Os05g09020.1
IIc	*EgWRKY82*	LOC105057329	1.926	1.21E-07	14	945	315	2	3				Nucleus	*OsWRKY03*	LOC_Os03g55080.1
IIc	*EgWRKY84*	LOC105037454	3.227	3.45E-10	1	873	291	0	1				Nucleus	*OsWRKY29*	LOC_Os07g02060.1
IId	*EgWRKY19*	LOC105039302	1.497	2.66E-11	2	948	316	2	3	1WRKYGQK	C-X_4-5_-C-X_22-23_-H-X_1_-H	C2H2	Nucleus	*OsWRKY51*	LOC_Os04g21950.1
IId	*EgWRKY45*	LOC105046551	-0.741	0.0003	6	1068	356	2	3				Nucleus	*OsWRKY94*	LOC_Os12g40570.2
IId	*EgWRKY63*	LOC105049350	1.085	0.0002	7	972	324	2	3				Nucleus	*OsWRKY51*	LOC_Os04g21950.1
IId	*EgWRKY65*	LOC105050398	2.832	4.58E-25	8	924	308	2	3				Nucleus	*OsWRKY51*	LOC_Os04g21950.1
IId	*EgWRKY69*	LOC105050834	0.705	0.0005	8	963	321	2	3				Nucleus	*OsWRKY51*	LOC_Os04g21950.1
IId	*EgWRKY77*	LOC105053433	1.829	9.78E-18	10	1062	354	2	3				Nucleus	*OsWRKY121*	LOC_Os03g53050.1
IIe	*EgWRKY27*	LOC105040793	3.867	7.77E-66	3	1122	374	2	3				Nucleus	*OsWRKY39*	LOC_Os02g16540.1
IIe	*EgWRKY50*	LOC105047488	-1.355	0.0425	6	831	277	2	3				Nucleus	*OsWRKY14*	LOC_Os01g53040.1
IIe	*EgWRKY58*	LOC105048100	-2.264	8.39E-15	7	861	287	2	3				Nucleus	*OsWRKY13*	LOC_Os01g54600.1
IIe	*EgWRKY60*	LOC105048265	2.811	3.86E-06	7	1113	371	2	3				Nucleus	*OsWRKY39*	LOC_Os02g16540.1
III	*EgWRKY07*	LOC105039486	4.147	2.33E-25	1	1068	356	2	3	1WRKYGQK	C-X_7_-C-X_23_-H-X_1_-C	C2HC	Nucleus	*OsWRKY69*	LOC_Os08g29660.1
III	*EgWRKY26*	LOC105040720	3.708	1.55E-72	3	1119	373	2	3				Nucleus	*OsWRKY69*	LOC_Os08g29660.1
III	*EgWRKY40*	LOC105045992	1.567	0.0001	5	930	310	2	3				Nucleus	*OsWRKY82*	LOC_Os05g14370.1
III	*EgWRKY52*	LOC105047564	5.854	1.07E-138	6	1080	360	0	1				Nucleus	*OsWRKY69*	LOC_Os08g29660.1
III	*EgWRKY59*	LOC105048220	4.916	1.14E-89	7	1092	364	2	3				Nucleus	*OsWRKY113*	LOC_Os06g06360.1
III	*EgWRKY73*	LOC105052308	1.327	0.0005	10	936	312	2	3				Nucleus	*OsWRKY45*	LOC_Os05g25770.1
III	*EgWRKY81*	LOC105057372	3.835	0.0003	14	1155	385	0	1				Nucleus	*OsWRKY69*	LOC_Os08g29660.1
IV	*EgWRKY29*	LOC105042138	-1.015	0.0198	3	594	198	1	2	1WRKYGQK	NA	NA	Nucleus	*OsWRKY72*	LOC_Os11g29870.1

Gene name was assigned based on by Xiao et al. [25]. Gene locus was determined based on oil palm reference genome accession number PRJNA192219 deposited in NCBI. Gene’s boundaries of exons and introns was determined using the GSDS2.0 (http://gsds.gao-lab.org/). Conserved domains analysis of EgWRKY amino acid sequences were identified using CDD database (http://www.ncbi.nlm.nih.gov/cdd/). Subcellular localization prediction was conducted using WoLF PSORT (https://wolfpsort.hgc.jp/). Rice orthologous genes identified using BLASTP search with default parameters and top hit was selected based on the alignment result (http://rice.plantbiology.msu.edu/analyses_search_blast.shtml).

**Fig. 2:**
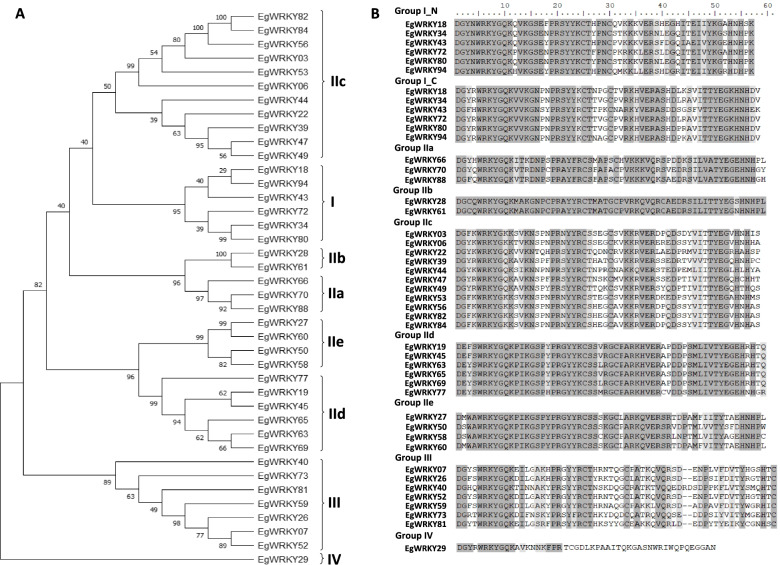
Phylogenetic analysis and multiple alignment analysis of 40 EgWRKY proteins. **A** The phylogenetic tree was constructed with MEGA X using UPGMA method based on protein sequences of WRKY domains found in the 40 EgWRKYs which were aligned using Clustal W prior to phylogenetic tree construction using MEGA X based on UPGMA method using Jones-Taylor-Thronton (JTT) substitution model and partial deletion method with 1000 bootstrap value. **B** Multiple sequence alignment of conserved WRKY domain in 40 EgWRKY proteins was performed using Clustal W programme. Conserved WRKY motif and zinc-finger pattern are indicated within Group or Subgroups with dark grey represents 100% sequence identity. Groups I_N and I_C indicate the N-terminus and C-terminus of the WRKY domain of Group I EgWRKY protein

The 40 *EgWRKY* genes were classified based on the number of conserved heptapeptide and zinc finger pattern in their protein sequences (Table 1). Among the 40 *EgWRKY* genes, 6 *EgWRKY* genes were categorized into Group I, 26 *EgWRKY* genes belong to Group II, 7 *EgWRKY* genes in Group III and only *EgWRKY29* were categorized in Group IV. Group II was sub-divided into 5 subgroups according to the unique pattern of the WRKY domain. Group IIc consisted of the highest number of *EgWRKY* genes among all groups with 11 members that possess either the common conserved heptapeptide pattern, WRKYGQK or a specific conserved heptapeptide patterns, WRKYGKK. The phylogenetic tree depicts the relationship and structural diversity among the 40 *EgWRKY* genes (Fig. 2A) in the 8 clades representing the 3 main Groups (I, III and IV) and 5 Subgroups (IIa-e), that are similar with the grouping based on the conserved domains (Fig. 2B). EgWRKY29 protein which does not have the zinc finger motif in the WRKY domain was classified in Group IV. Members in Group I showed closer relationship with the members in Subgroup IIc. Multiple sequence alignment of conserved WRKY domain in the 40 EgWRKY proteins was performed to study the variation among and within the Groups or Subgroups (Fig. 2B). Two members in Subgroup IIb i.e., EgWRKY28 and 61 were 98.3% identical to each other. Members within other Subgroups also showed higher sequence identity (more than 80%) except for Subgroup IIc while the lowest sequence similarity was observed among the members within Group III (50%).

**Fig. 3:**
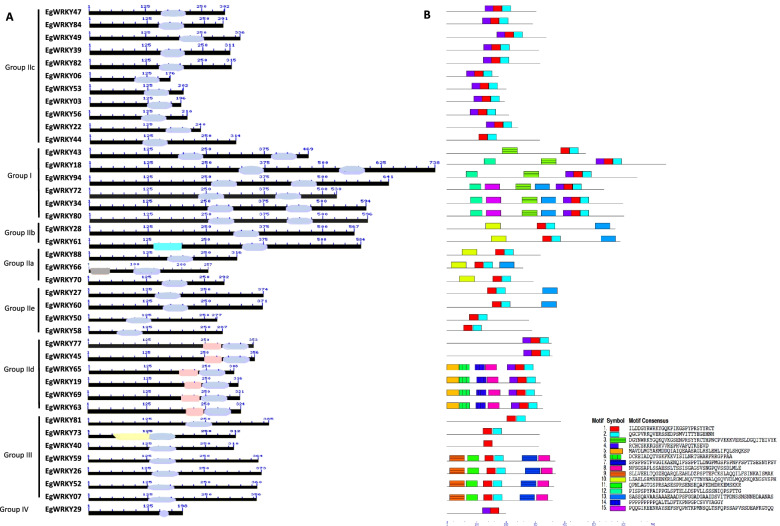
Illustration of conserved domains and conserved motifs identified in 40 EgWRKY proteins. **A** Identification of conserved domains was performed by searching against conserved domain database (CDD). Four conserved domains, bZIP (grey), uncharacterized conserved domain COG4372 (cyan), Plant Zn cluster (pink), ligand-dependent nuclear receptor-interacting factor 1 (LRIF) domain (yellow) and WRKY (blue) identified in EgWRKY proteins. **B** Conserved motifs were detected using Multiple Em for Motif Elicitation (MEME) software. Each motif with conserved amino acid residues is represented in different colour (motif 1 – 15) as shown in lower panel

We further identified the conserved domains in the EgWRKY amino acid sequences using the CDD database. The complete WRKY domain was observed in 39 EgWRKY proteins except for EgWRKY29 in which lacks the zinc finger motif (Fig. 3A). We noticed the presence of plant zinc cluster domain (40 residues) associated with the WRKY domain in all the members of Subgroup IId. Basic leucine zipper (bZIP) domain (70 residues) involved in DNA-binding and dimerization was found in EgWRKY66 of Subgroup IIa. EgWRKY61 protein contains an uncharacterized conserved domain (COG4372) with unknown function while EgWRKY73 protein has a ligand-dependent nuclear receptor-interacting factor 1 (LRIF) domain. Fifteen motifs specific to EgWRKY proteins were identified (Fig. 3B). Motif 1 which comprised of a heptapeptide WRKYG[Q/K]K was widely distributed in all 40 EgWRKY proteins. We observed that the distribution patterns of the motifs were specific to Group or Subgroups, for instance, motifs 3 and 15 were found only in Group I, motif 5 in Subgroup IId and motif 9 in Subgroup IId.

### *EgWRKY* genes are enriched with light-, stress- and hormone-responsive elements in their promoters

**Fig. 4:**
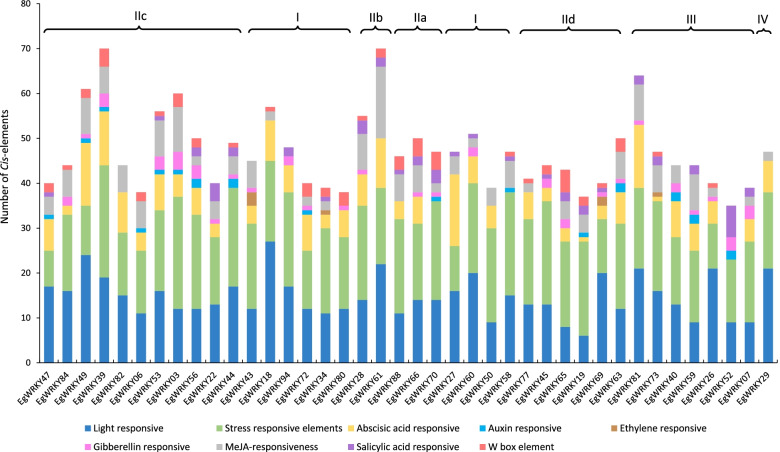
Promoter sequence analysis of 40 *EgWRKY* genes responsive to drought stress arranged according to their grouping. **A** *In-silico* analysis of *cis*-regulatory elements in the promoter regions (2.0 kb upstream) of targeted *EgWRKY* genes by using PlantCARE

To identify the *cis*-regulatory elements corresponding to stress response and hormone signalling, we performed *in-silico* analysis of *cis*-regulatory elements in the 2 kb upstream sequence which covers both the 5’-UTR and promoter of the targeted *EgWRKY* genes. Light-responsive elements (GT1-motif, G-box and Sp1 element) and stress-responsive elements (dehydrin-responsive element, DRE; F-box; *cis*-acting element involved in low-temperature responsiveness, LTR) were predicted and found to be significantly abundant in the promoter regions of all the 40 differentially expressed *EgWRKY* genes (Fig. 4A). The highest number of light responsive elements (27 binding sites) was found in the promoter of *EgWRKY18* while the highest number of stress-responsive elements (25 sites) was predicted in the promoters of *EgWRKY39* and 0*3*. The stress-responsive elements including MBS (MYB TF binding site involved in drought inducibility), LTR and TC-rich repeats (cis-acting element involved in defence and stress responsiveness) were found in the promoter of *EgWRKY84*. We also found W box element in the promoter of 27 *EgWRKY* genes and *EgWRKY65* has the highest number i.e. 5 copies of W box elements, among the 40 *EgWRKY* genes. The ABA-responsive elements were predicted in the promoter of 36 *EgWRKY* genes except *EgWRKY44, 70, 58* and *5,2* and the highest number (i.e., 16) of binding sites were found in *EgWRKY27*. Other hormone-responsive elements like MeJA-responsive (16 in *EgWRKY61*), salicylic acid-responsive (7 in *EgWRKY52*) and gibberellin-responsive (4 in *EgWRKY03*) were also observed in the promoter of *EgWRKY* genes.

### Drought responsive DEG *EgWRKYs* are involved in different growth developmental stages

**Fig. 5:**
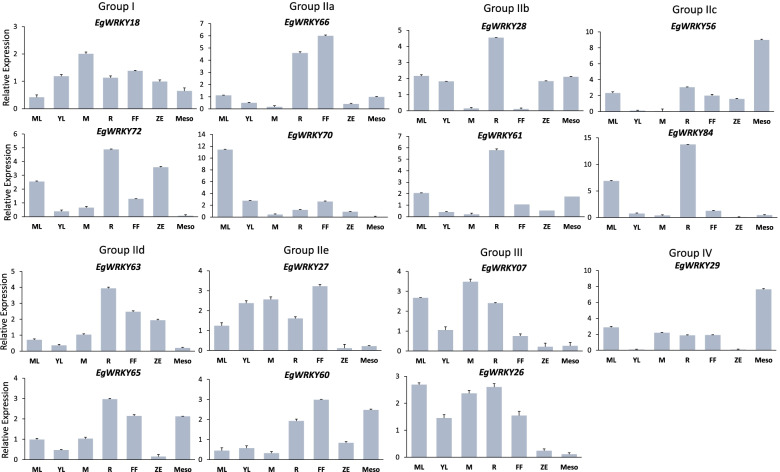
Expression profiles of selected *EgWRKY* in different tissues. Three housekeeping genes (*EgGRAS*, *EgCyp2* and *EgSLU7*) were used as internal reference genes. ML: mature leaf, YL: young leaf, M: meristem, R: root, FF, female inflorescence, ZE: zygotic embryo, Meso: mesocarp. Each value is the mean ± SE of 3 technical triplicates

To gain insight into the roles of Dro-*EgWRKY* at different stages of plant growth and development, we examined the expression profile of two randomly selected *EgWRKY* members from each group in the mature leaf, young leaf, meristem, root, female inflorescence, zygotic embryo and mesocarp tissues. We noticed a similar expression trend in the members of Group IIb (*EgWRKY28* and *61*) and Group III (*EgWRKY07* and *26*) while the rest of the groups exhibited different tissue specific expression pattern (Fig. 5). Both *EgWRKY28* and *61* from Group IIb were highly expressed in the root followed by mature leaf and mesocarp samples. *EgWRKY07* and *EgWRKY26* from Group III were expressed predominantly in the vegetative tissues including mature leaf, young leaf, meristem and root. Among the 15 selected *EgWRKYs*, 8 of them (*EgWRKY07, 18*, *26, 27, 60*, *61, 63* and *65*) were expressed in all seven tissues tested while the remaining seven genes had relatively low expression in some tissues.

### Drought responsive DEG *EgWRKYs* are responsive to other abiotic stresses, hormones and H_2_O_2_ treatments

**Fig. 6:**
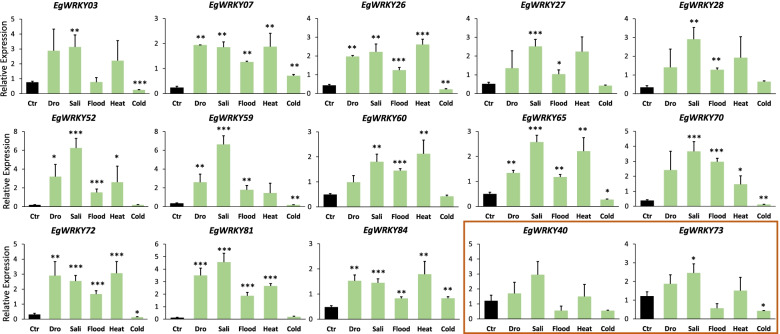
Expression profiles of selected *EgWRKY* under different abiotic stress treatments (green) and control (black). Additional candidates genes from Group III that were analyzed are boxed in brown. Three housekeeping genes (*EgGRAS*, *EgCyp2* and *EgSLU7*) were used as internal reference genes. Each value is the mean ± SE of 3 independent biological replicates. Asterisks indicate significant differences between each treatment point and controls (*P < 0.05, **P < 0.01, ***P ≤ 0.001, Student’s t-tests)

To evaluate the transcript abundance of *EgWRKY*s in response to different abiotic stresses, we examined the gene expression of 13 upregulated drought responsive *EgWRKY* with the highest fold change of differential expression identified from RNA-Seq, in cold, drought, flood, heat and salinity treated samples (Fig. 6). Among 13 *EgWRKY* genes, 5 genes were from Group III, 2 genes each from Subgroup IIc and e while 1 gene each from Group I, Subgroup IIa, b and c. Coincidently, the majority of the highly differentially expressed *EgWRKYs*, which were expressed higher than 3.7-fold under drought condition were from Group III, hence, the remaining two members *EgWRKY40* and *73* from the same group with lower differential gene expression (1.3-1.5-fold) were also examined. Among the 15 *EgWRKY* genes analysed, 7 of them (*EgWRKY07*, *26*, *52*, *65*, *72*, *81* and *84*) were significantly upregulated by at least 1.3-fold to as high as 6.3-fold by drought, salinity, flood and heat treatments. However, they were significantly downregulated by cold treatment except for *EgWRKY07* and *84* whereby they were significantly upregulated by cold treatment. Besides, *EgWRKY28*, *59, 60* and *70* were significantly upregulated by salinity and flood from 1.3-fold to 6.6-fold. Both *EgWRKY03* and *73* were significantly upregulated by salinity treatment but downregulated by cold treatment. No significant differentially gene expression observed in *EgWRKY40*. Hence, apart from *EgWRKY40*, all 14 genes being analysed were significantly differentially expressed in all abiotic stress treatments and may be serve as potential stress-responsive candidates for further studies.

**Fig. 7:**
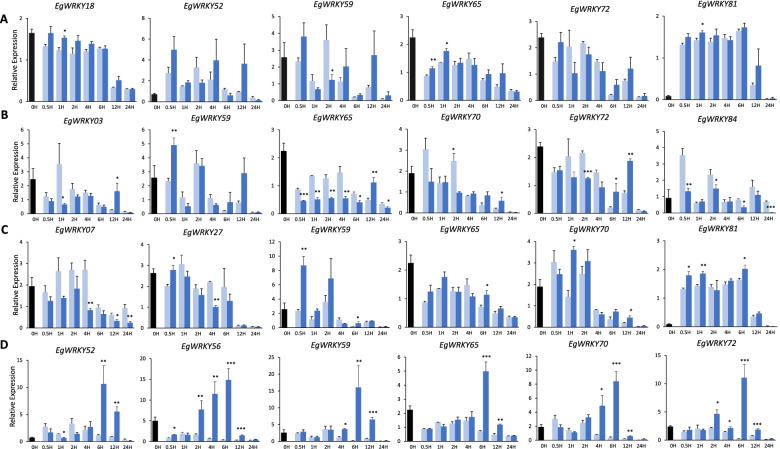
Expression profiles of selected *EgWRKY* under different phytohormones treatments for 24 hours. Leaf incubated on MS media supplement with 100 μM ABA (**A**), 100 μM MeJA (**B**), 100 μM SA (**C**) and 10 mM H_2_O_2_ (**D**) were sampled at different time points (0h, 0.5h, 2h, 4h, 6h, 12h, 24h). Three housekeeping genes (*EgGRAS*, *EgCyp2* and *EgSLU7*) were used as internal reference genes. Each value is the mean ± SE of 3 independent biological replicates. Black bar indicates control which not treated at 0h, light blue and blue bars indicate control and treated samples, respectively at the treatment time. Asterisks indicate significant differences between each treatment point and controls (*P < 0.05, **P < 0.01, ***P ≤ 0.001, Student’s t-tests)

Since hormones and H_2_O_2_ are signalling molecules for plant stress responses and we identified various hormones-responsive elements in the promoters of several drought responsive *EgWRKYs* TFs, we further conducted hormones (ABA, MeJA and SA) and H_2_O_2_ treatments using young leaves to study their effects on the expression of drought responsive *EgWRKY* genes, harbouring the respective elements in their promoter (Fig. 7). For ABA treatment, *EgWRKY18* and *81* with 9 and 14 ABRE elements, respectively were significantly upregulated at 1.5-fold and 1.2-fold, respectively after 1h of treatment (Fig. 7A). *EgWRKY65* gene with 3 ABRE elements showed significant up-regulation after 30min and 1h post treatment at 1.1-fold and 1.7-fold, respectively. However, *EgWRKY59* was significantly downregulated after 2h of treatment. In MeJA treatment, *EgWRKY59* was significantly upregulated at 5-fold after 30min of exposure while we observed significant downregulation of *EgWRKY03, 65* and *84* at various time points (Fig. 7B). Interestingly, both *EgWRKY70* and *72* were downregulated after 2h of exposure, but they were upregulated at 6h time point (Fig. 7B). For SA treatment, both *EgWRKY59* and *81* were upregulated at different expression level after 30min, 1h and 6h of exposure (Fig. 7C). *EgWRKY*27, *65* and *70* were significantly upregulated at various time points at different expression level. Then, *EgWRKY07* and 27 were downregulated at different expression level after 4h of exposure. In H_2_O_2_ treatment, the expression of all six selected *EgWRKY52*, *56*, *59*, *65*, *70* and *72* genes were significantly upregulated after 6 h of exposure and the highest expression was observed in *EgWRKY56* and *59* at more than 10-fold higher compared to control (Fig. 7D). Among them, *EgWRKY56* was more sensitive to H_2_O_2_ treatment as indicated by early significant positive response after 30min of exposure at 1.6-fold and its expression was significantly upregulated at all time points except for 1h. Besides at 6h time point, *EgWRKY59, 70* and *72* were also significantly upregulated at different expression level after 4h of exposure. Collectively, the findings provide evidence that the selected *EgWRKY* genes were responsive to different hormonal treatments and highly responsive to H_2_O_2_ treatment at different scale of expression level.

## Discussion

WRKY TFs demonstrate dynamic roles in many aspects of plant life cycle including plant development [9, 10], nutrient uptake [13], biotic stress [21, 28] and abiotic stress [18, 27, 29–31]. Extensive studies report that WRKY TFs are potent regulators in conferring drought tolerance in plants [26, 29, 32–37], modulated by phytohormones especially ABA [35]. Oil palm yield is severely affected by drought stress resulting in low flower sex ratio and early fruit bunch abortion [24] which cause huge economic losses. Nevertheless, little is known about the functions of WRKY TFs in drought response in oil palm. Hence, it is crucial to identify *EgWRKY* TFs that may improve drought tolerance in oil palm.

In this study, we identified 40 differentially expressed *EgWRKY* TFs responsive to drought stress in oil palm leaf using RNA-seq. We further evaluated their response to different abiotic stresses and phytohormone treatments. Our RNA-seq results revealed that the *WRKY* TFs were among the TFs that were responsive to drought stress in oil palm with 80% of them upregulated by drought stress, followed by TFs in the *AP2-EREBP*, *NAC* and *bZIP* families. Further characterization of the *EgWRKY* candidates in this study will shed light on their possible roles in drought response in oil palm.

A total of 95 *WRKY* TFs were identified in the oil palm genome by Xiao et al. [25]. They were named according to the chromosomal location and classified into 8 groups based on their phylogenetic relationship [25], instead of following the general classification of WRKY TFs into 3 main Groups I, II and III, with 5 Subgroups in Group II (IIa to IIe) [4]. In our study, we categorized 40 drought responsive *EgWRKY* DEGs into 4 groups (Groups I, IIa-e, III and IV) according to the general classification method to ease the comparison with WRKY TFs from other plants. Phylogenetic analysis revealed the close relationship of *EgWRKY* members in Group I and Group II that are represented by 4 clades. Notably, *EgWRKY29* was categorized into Group IV due to the absence of an intact zinc finger motif in its WRKY domain, which may be due to a deletion event. In concordance, the presence of Group IV WRKY TFs which lack an intact zinc-finger motif in the WRKY domain is also reported in plants such as *Pennisetum glaucum* [30] and *Ammopiptanthus nanus* [38]. We have successfully assigned these 40 drought responsive *EgWRKYs* to oil palm CHR including *EgWRKY84*, *88* and *94* genes that could not be mapped to the oil palm reference genome by Xiao et al. [25]. They were now successfully mapped to CHR1, 2 and 15, respectively; using the improved genome assembly [19]. To ease future referencing, we maintained the nomenclature of *EgWRKY84*, *88* and *94* regardless of their chromosomal locations. It is worth mentioning that the Dro-*WRKY* TFs were mainly found on CHR3, 5, 6 and 7 which constituted 50% of the total Dro-*WRKY* TFs. CHR7 harbours 6 WRKYs of which 5 were upregulated by drought among 9 WRKYs located in CHR7. Hence, this information serves as a good reference for the subsequent effort in identifying drought hotspots in oil palm genome through mapping of the drought responsive DEGs and drought responsive markers discovered in oil palm [39].

To understand the functions of the Dro-*EgWRKY*s, we first profiled their tissue-specific gene expression, subsequently their response to various abiotic stress and phytohormone treatments. WRKY TFs are involved in different developmental stages of plants such as adventitious root formation [9], leaf senescence and flowering [10]. Here, we noticed that *EgWRKY70* and *84* were preferentially expressed in mature leaf, both *EgWRK29* and *56* were predominantly expressed in mesocarp while *EgWRKY61* and *84* were detected abundantly in root tissue, suggesting their possible involvement in different stages of oil palm growth and development. This tissue-specific expression profile is not associated with their grouping as there was no specific expression pattern observed among the members from the same group. However, it must be further validated by analysing more members of same group instead of only 2 members from each group. During the onset of drought stress, root is the first organ that responses to water stress signal while leaf responds to drought stress through stomatal closure [40]. Both *EgWRKY61* (Group IIb) and *84* (Group IIc) were highly expressed in the mature leaf and root tissues, indicating their possible roles in responding to water stress signal in both organs during drought stress. *WRKY* TFs were reported to regulate transcription of stress related genes that respond to multiple abiotic stresses in particular drought and salinity as both shared similar signal transduction pathway [41]. In *Arabidopsis*, *AtWRKY46* was upregulated by drought, salinity, SA and H_2_O_2_ treatments [18]. *GhWRKY41* [16] from *G. hirsutum* were upregulated by drought and salt stress in the transgenic *N. benthamiana* by removing ROS to better adjust the osmotic stress in a ABA-dependent manner [19]. In oil palm, 13 selected *EgWRKYs* were found to be significantly expressed in samples treated with different abiotic stresses, especially *EgWRKY26*, *65*, *72* and *81* that exhibited a significant increase in expression levels in response to drought, heat and salinity. These 4 candidates were also identified as DEGs in oil palm under salinity and heat stresses (unpublished data). Collectively, these results suggest that the drought responsive Eg*WRKY* TFs may be involved in the regulation of multiple abiotic stress responses, possibly sharing the same mechanism in responding to environmental stimuli to induce stress related genes.

Much attention has been channelled to study the functions of Group III members of WRKY TFs due to their involvement in multiple processes from plant development to stress signalling response [42–44]. The Group III members are distinguishable from other Group members by the presence of zinc finger type C2HC instead of C2H2. Herein, we validated that the expression levels of all 7 members of Group III WRKYs including *EgWRKY07*, *26*, *40*, *52*, *59*, *73* and *81* were upregulated by drought stress in oil palm seedlings. We also showed that they were upregulated by other abiotic stresses including cold, flood, heat and salinity. In *G. hirsutum,* the Group III *WRKY* members including *GhWRKY31*, *59* and *102* were also involved in fibre development and leaf senescence apart from abiotic stress response [43]. Furthermore, many Group III members of WRKY TFs have also been reported to be involved in biotic stress response. For instance, six members of the Group III WRKY TFs from *Solanum lycopersicum* were involved in the tomato yellow leaf curly virus (TYLCV) defence signalling pathway [45], and *ScWRKY5,* a Group III *WRKY* gene from sugarcane was upregulated by fungal infection, drought, salinity and hormonal stresses [44]. We observed a relatively higher gene expression levels of two Group III *WRKY* members (*EgWRKY07* and *26*) in the oil palm vegetative tissues including mature leaf, young leaf and root. This suggests that some Group III drought responsive *EgWRKY* TFs may play multiple roles in abiotic stress response, growth and development in oil palm. In addition, further analysis on the sequences of *EgWRKY* TFs in oil palm revealed a total of 13 *EgWRKY* TFs from Group III (unpublished data). Hence, further investigation on the remaining 6 members of Group III *EgWRKY* TFs may provide a clear indication of the functions of Group III *EgWRKY* TFs in oil palm.

In plants, phytohormones play crucial roles in controlling physiological responses to environmental stimuli especially ABA, SA, MeJA and ET *via* transcriptional modulation of transcription factor genes and stress responsive genes [15, 36, 46]. ABA is known as a stress hormone as it positively regulates plant responses to different environmental stresses particularly drought stress by triggering stomatal closure to reduce the transpirational water loss in leaf during water scarcity [47]. Besides, both SA and MeJA are also involved in attuning plant responses to different abiotic stresses through interaction with ABA, mediated by TFs including MYC, NAC and WRKY [47, 48]. Studies have shown that WRKY TFs regulate plant responses to different stresses under the influence of phytohormones including ABA, SA, MeJA and ET [49]. This subsequently leads to the activation of stress-responsive genes to confer tolerance to bacteria pathogen [50], fungal pathogen [21] and to abiotic stresses like drought, salinity, heat and cold [16, 17]. In the current study, we examined the effects of ABA, SA and MeJA on the gene expression levels of drought responsive *EgWRKY* genes harbouring the corresponding *cis*-elements in their promoter. We provide evidence that both *EgWRKY65* and *81* were upregulated by drought, heat, salinity, flood stresses and hormonal treatments which included ABA and SA at certain time points. These results suggest a crosstalk between ABA and SA in the signalling transduction to trigger response against different abiotic stresses which is in agreement with *ZmWRKY40* from maize [51]. Interestingly, *EgWRKY59* gene (a Group III member) was significantly upregulated by drought, heat, flood and salinity stresses, was also found to be significantly upregulated by three phytohormones including ABA, MeJA and SA at certain time points, suggesting the potential involvement of *EgWRY59* in the regulatory network of three abiotic stresses via crosstalks of three phytohormones. Hence, *EgWRKY59* is one of the potential candidates for further characterization as abiotic stress markers in oil palm.

During the onset of abiotic stress, excessive ROS (H_2_O_2,_ superoxide radicals, hydroxyl radical) are generated that cause oxidative stress to plants and subsequently inhibit normal growth and reproduction in plants [52]. Plants develop different defence systems to counter oxidative stress through the activation of ROS scavenging enzymes (superoxide dismutase and peroxidases), regulation of the downstream stress response genes and accumulation of osmoprotectants such as proline, glycine and trehalose [53, 54]. Here, we examined the expression levels of drought responsive *EgWRKYs* in response to high oxidative stress induced by H_2_O_2_ treatment. All 6 *EgWRKYs (52, 56, 59, 65, 70* and *72*) were highly sensitive to H_2_O_2_ treatment with upregulation of transcript levels after 6h of treatment. This was also observed in the expression profiles of *Aquilaria. sinensis AsWRKY* genes that were upregulated upon H_2_O_2_ treatment, in particular *AsWRKY25* that increased 100-fold in gene expression after 12h of treatment [55]. In addition, many studies reported the involvement of *WRKY* TFs in reducing oxidative stress by controlling the stomatal closure mediated by ABA and SA [15, 36, 56]. Here, we found that *EgWRKY59* and *EgWRKY65* were upregulated by ABA and SA treatments, and they were also significantly upregulated by drought, heat, flood and salinity treatments in oil palm. These findings collectively suggest a crosstalk between H_2_O_2_ and phytohormones (ABA, SA) in regulating the gene expression of *EgWRKY59* and *EgWRKY65* in adaptation to the increase in ROS level, probably through the transcription regulation of genes encoding ROS scavenger. Further characterization is required to provide more evidence to support this observation.

## Conclusion

A total of 40 DEGs encoding *WRKY* TFs were identified from the transcriptome of drought treated leaf of oil palm. All 32 upregulated Dro-*EgWRKY* genes have preferential expression in different tissues, and exhibited different response to abiotic stresses, phytohormones and H_2_O_2_ treatments. *EgWRKY59* and *EgWRKY65* may share similar regulatory mechanism involving ABA-, SA- and ROS-mediated signalling during drought and other abiotic stresses and are potential candidate genes for conferring higher tolerance to these types of stress. *EgWRKY07, 26, 59* and *EgWRKY81 from* Group III may involve in the regulation of different abiotic stress responses. Further functional studies of these candidate genes are required to evaluate their potential as drought biomarker to screen for oil palm with better drought tolerance and as candidate genes for genetic improvement purpose.

## Methods & materials

### Plant materials, growth conditions and treatments

Mature leaf, young leaf, female inflorescence, zygotic embryo and mesocarp were sampled from 15-year-old commercial DxP (Deli Dura x Pisifera) palm. Meanwhile, meristem and root were sampled from 1-year-old DxP (Deli Dura x Pisifera) seedling. For different abiotic stress treatments, 6-month-old oil palm Dura (Deli Dura) seedlings planted in polybags filled with top soil were acclimatized in greenhouse environment for 1 month at 28 °C prior to exposure to abiotic stress treatments for two weeks at 28 °C except for the cold and heat treatments. Six biological replicates of oil palm seedlings were used for each stress treatment. Three independent oil palm seedlings and three untreated oil palm seedlings (controls) were randomly selected for RNA-Seq study. For control, seedlings were watered daily with 200 mL of tap water. For seedlings in the drought treatment, no watering was conducted for two weeks. For flood treatment, the water level was maintained one inch above the soil level. In salinity treatment, seedlings were irrigated with 200 mL of 200 mM NaCl daily. Seedlings were incubated at 15 °C and 35 °C while watering remain the same as control seedling for cold and heat treatment, respectively. Hormonal and H_2_O_2_ treatments were conducted on the young leaf pieces of 2 x 2 cm dimension incubated on MS agar plate supplemented with 100 μM ABA, 100 μM MeJA, 100 μM SA, 10 mM H_2_O_2_ for a total incubation period of 6h. Four leaf pieces were sampled at 0, 0.5, 2, 4 6, 12 and 24h.

### RNA-Seq analysis

Total RNA of oil palm leaves was isolated from 3 control and 3 drought treated oil palm seedlings sharing the same parents using MN Nucleospin RNA Plant Kit (Macherey-Nagel, Germany) according to the manufacturer's instructions. The quality and concentration of total RNA samples were evaluated using high sensitivity Bioanalyzer chip (Agilent Technologies, USA) prior to library preparation. Sequencing and bioinformatic analysis were carried out using Illumina HiSeq2000 (Novogene Bioinformatics Technology, China). After removing adaptor and low quality reads, clean reads were mapped onto oil palm reference genome accession number PRJNA192219 deposited in NCBI, using TopHat2 algorithm with a maximum mismatch set at 2 [57]. Gene expression level was quantified using fragments per kilobase of transcript over million mapped reads (FPKM) method and analysed using HTSeq software [58]. Subsequent identification of DEGs was performed using DESeq software with a corrected *p*-values < 0.05 [59]. TF analysis on the DEGs was conducted using iTAK program V1.2 according to default parameters [60].

### Sequence analysis

Gene and protein sequences of the Dro-*EgWRKY* candidates identified from RNA-Seq were retrieved from National Center for Biotechnology Information (NCBI) and matched with the sequences reported by Xiao et al. [25] by using BLAST-N and BLAST-P tools at default setting. Protein sequences of WRKY domain found in the EgWRKYs were aligned using Clustal W prior to phylogenetic tree construction using MEGA X based on UPGMA method using Jones-Taylor-Thronton (JTT) substitution model and partial deletion method with 1000 bootstrap value. . Conserved domains in EgWRKY amino acid sequences were identified using CDD database (http://www.ncbi.nlm.nih.gov/cdd/). The conserved motifs in the full length EgWRKY proteins were analysed using the Multiple Em for Motif Elicitation (MEME) program (https://meme-suite.org/meme/tools/meme). MEME motifs are represented by position-dependent letter-probability matrices which describe the probability of possible letter at each position in the pattern. The maximum number of motifs was set at 15, the maximum motif length was set at 60 amino acids, the optimum motif width was restricted at 6 to 300 residues, and the other default parameters were used. Subcellular localization prediction was conducted using WoLF PSORT (https://wolfpsort.hgc.jp/). Identification of rice ortholog genes was performed using BLASTP search with default parameters and top hit was selected based on the alignment result (http://rice.plantbiology.msu.edu/analyses_search_blast.shtml). Identification of cis-regulatory elements in the putative 5’-UTR and promoter regions of the targeted EgWRKY genes was conducted on the 2 kb upstream sequence from the start codon of genomic sequence using PlantCARE (http://bioinformatics.psb.ugent.be/webtools/plantcare/html/).

### Quantitative RT-PCR (qRT-PCR) analysis of transcripts

The first-strand cDNA was synthesized from total RNA of oil palm tissue using Maxima First Strand cDNA Synthesis Kit (Thermo Scientific, USA) and quantified using StepOne Plus (Applied Biosystems, USA) and Fast SYBR Green Master Mix (Applied Biosystems, USA) according to the manufacturer’s instructions. Dissociation curves were generated to verify the amplification specificity. Independent qRT-PCR runs were conducted in technical triplicates for different tissues and both biological and technical triplicates for abiotic stress, hormonal and H_2_O_2_ treatments and the calibrated normalized relative quantity (CNRQ) values of the transcripts were calculated using delta–delta Ct method [61]. Expression of target genes in oil palm mesocarp were normalized to *Gibberellin-responsive protein 2 (EgGRAS*), *cyclophilin 2* (*EgCyp2*) and *Pre-mRNA splicing factor SLU7* (*EgSLU7*) [62]. Student’s t-test was conducted using the log_2_ value of relative expression level to evaluate the statistical significance in the differences observed in target gene expression between the control and treated samples.

## Supplementary Information

**Additional file 1: Table S1.** Expression data of differential expressed genes (DEGs) encode for APE-EREBP transcription factors. **Table S2.** Expression data of differential expressed genes (DEGs) encode for bHLH transcription factors. **Table S3.** Expression data of differential expressed genes (DEGs) encode for bZIP transcription factors. **Table S4.** Expression data of differential expressed genes (DEGs) encode for C2H2 transcription factors. **Table S5.** Expression data of differential expressed genes (DEGs) encode for MYB transcription factors. **Table S6.** Expression data of differential expressed genes (DEGs) encode for NAC transcription factors. **Table S7.** Expression data of differential expressed genes (DEGs) encode for Orphans transcription factors. **Table S8.** Expression data of differential expressed genes (DEGs) encode for SBP transcription factors.

**Additional file 2: Table S9.** qRT-PCR primers

**Additional file 3.** Promoter sequences of 40 Dro-EgWRKY genes used in the promoter sequence analysis (Fig. 4) .

## Data Availability

The reference genome assembly used for data analysis was obtained from National Center for Biotechnology Information (NCBI) BioProject PRJNA192219. The raw transcriptome data generated and analysed in this study deposited in SRA of the NCBI under accession number PRJNA775831. All data used for the phylogenetic tree analysis can be accessed from Treebase at http://purl.org/phylo/treebase/phylows/study/TB2:S29059. The datasets analysed during this study are included in this published article and its supplementary information files.

## Abbreviations

ABA: Abscisic acid
ABF3: ABA-response element binding factor 3
bZIP: Basic leucine zipper
CHR: Chromosome
DEGs: Differentially expressed genes
Dro-*EgWRKY*: Drought responsive oil palm *WRKY*
ET: Ethylene
FPKM: Fragments per kilobase of transcript over million mapped reads
H_2_O_2_: Hydrogen peroxide
MeJA: Methyl jasmonate Oil palm *WRKY * *EgWRKY*
Oil palm *WRKY*: *EgWRKY*
qRT-PCR: Quantitative RT-PCR
SA: Salicylic acid
TYLCV: Tomato yellow leaf curly virus
TFs: Transcription factors
