# Supplementary material for: Identification of drought responsive Elaeis guineensis WRKY transcription factors with sensitivity to other abiotic stresses and hormone treatments
Source: BMC Genomics. 2022 Feb 26;23:164. doi: 10.1186/s12864-022-08378-y (PMC8882277; doi:10.1186/s12864-022-08378-y)
Supplement: Supplementary file 1 — Additional file 1: Table S1. Expression data of differential expressed genes (DEGs) encode for APE-EREBP transcription factors. Table S2. Expression data of differential expressed genes (DEGs) encode for bHLH transcription factors. Table S3. Expression data of differential expressed genes (DEGs) encode for bZIP transcription factors. Table S4. Expression data of differential expressed genes (DEGs) encode for C2H2 transcription factors. Table S5. Expression data of differential expressed genes (DEGs) encode for MYB transcription factors. Table S6. Expression data of differential expressed genes (DEGs) encode for NAC transcription factors. Table S7. Expression data of differential expressed genes (DEGs) encode for Orphans transcription factors. Table S8. Expression data of differential expressed genes (DEGs) encode for SBP transcription factors. [file 12864_2022_8378_MOESM1_ESM.pdf]

Table S1 Expression data of differential expressed genes (DEGs) encode for APE-EREBP transcription factors.

No	Gene Locus ID	log2FoldChange (Dro vs Ctrl)	Up-Down expression	Padj	KEGG Annot Info	Description
1	105056761	8.3996	Up	3.98E-32	ethylene-responsive transcription factor ERF017	ethylene-responsive transcription factor ERF017-like
2	105059333	7.5981	Up	2.86E-17	dehydration-responsive element-binding protein A-4	ethylene-responsive transcription factor ERF027-like
3	105054150	6.9587	Up	1.16E-165	CBF4; dehydration-responsive element-binding protein 1D	dehydration-responsive element-binding protein 1G-like%2C transcript variant X3
4	105058987	6.143	Up	3.16E-13	CBF2; dehydration-responsive element-binding protein 1C; K09286 EREBP-like factor	dehydration-responsive element-binding protein 1F-like
5	105058243	6.0892	Up	5.47E-23	RRTF1; ethylene-responsive transcription factor ERF109	ethylene-responsive transcription factor ERF071-like
6	105043386	5.9478	Up	3.70E-136	CBF4; dehydration-responsive element-binding protein 1D	dehydration-responsive element-binding protein 1G
7	105060890	5.3602	Up	1.71E-85	RRTF1; ethylene-responsive transcription factor ERF109	ethylene-responsive transcription factor ERF109-like
8	105059334	5.2869	Up	6.11E-131	CBF2; dehydration-responsive element-binding protein 1C; K09286 EREBP-like factor	dehydration-responsive element-binding protein 1C
9	105054588	5.2093	Up	4.99E-92	CBF2; dehydration-responsive element-binding protein 1C; K09286 EREBP-like factor	dehydration-responsive element-binding protein 1C-like
10	105054362	4.898	Up	3.00E-05	ethylene-responsive transcription factor ERF025	--
11	105043387	4.719	Up	1.42E-15	ethylene-responsive transcription factor ERF025	ethylene-responsive transcription factor ERF027-like
12	105039334	4.0734	Up	7.88E-16	ethylene-responsive transcription factor ERF061	ethylene-responsive transcription factor ERF061-like
13	105049790	3.8817	Up	0.021939	ethylene-responsive transcription factor ERF019	ethylene-responsive transcription factor ERF020-like
14	105056759	3.8265	Up	2.20E-12	ORA47; ethylene-responsive transcription factor ERF018	ethylene-responsive transcription factor ERF018-like
15	105044742	3.6384	Up	3.24E-77	DREB2C; dehydration-responsive element-binding protein 2C	dehydration-responsive element-binding protein 2B-like%2C transcript variant X1
16	105041345	3.5541	Up	6.97E-13	ethylene-responsive transcription factor ERF012	ethylene-responsive transcription factor ERF014-like
17	105042035	3.4983	Up	7.25E-27	ethylene-responsive transcription factor ERF017	ethylene-responsive transcription factor ERF017-like%2C transcript variant X4
18	105035055	3.4932	Up	9.47E-29	ERF12; ethylene-responsive transcription factor 12	ethylene-responsive transcription factor 12-like
19	105039877	3.4275	Up	0.0004262	HRE2; ethylene-responsive transcription factor ERF071	ethylene-responsive transcription factor ERF114-like
20	105036666	3.3704	Up	3.08E-11	ethylene-responsive transcription factor ERF016	ethylene-responsive transcription factor ERF017-like
21	105053432	3.1338	Up	1.05E-23	ERF3; ethylene-responsive transcription factor 3; K09286 EREBP-like factor	ethylene-responsive transcription factor 3-like
22	105045154	3.075	Up	2.13E-57	HRE2; ethylene-responsive transcription factor ERF071	ethylene-responsive transcription factor ERF071-like
23	105050545	2.7379	Up	1.21E-06	ERF12; ethylene-responsive transcription factor 12	ethylene-responsive transcription factor 12
24	105035740	2.6634	Up	8.10E-17	ERF9; ethylene-responsive transcription factor 9; K09286 EREBP-like factor	ethylene-responsive transcription factor 4-like
25	105037298	2.548	Up	0.0011262	HRE2; ethylene-responsive transcription factor ERF071	ethylene-responsive transcription factor ERF071-like
26	105050553	2.3262	Up	3.78E-09	ERF9; ethylene-responsive transcription factor 9; K09286 EREBP-like factor	ethylene-responsive transcription factor 9
27	105043213	2.3133	Up	0.0020651	ERF2; ethylene-responsive transcription factor 2; K14517 ethylene-responsive transcription factor 2	pathogenesis-related genes transcriptional activator PTI5-like
28	105045153	2.26	Up	6.88E-16	EBP; ethylene-responsive transcription factor RAP2-3; K09286 EREBP-like factor	ethylene-responsive transcription factor RAP2-3-like
29	105040353	2.2122	Up	1.03E-10	WRI1; ethylene-responsive transcription factor WRI1	ethylene-responsive transcription factor WRI1-like
30	105050183	2.1864	Up	1.92E-23	ERF3; ethylene-responsive transcription factor 3; K09286 EREBP-like factor	ethylene-responsive transcription factor 3-like
31	105039637	2.1079	Up	0.035904	AIL5; AP2-like ethylene-responsive transcription factor AIL5; K09285 AP2-like factor, ANT lineage	AP2-like ethylene-responsive transcription factor AIL5
32	105048614	2.0931	Up	1.79E-16	RAP2.4; ethylene-responsive transcription factor RAP2-4; K09286 EREBP-like factor	ethylene-responsive transcription factor ERF060-like
33	105033078	2.0527	Up	1.03E-06	ERF3; ethylene-responsive transcription factor 3; K09286 EREBP-like factor	ethylene-responsive transcription factor 12-like
34	105050144	2.019	Up	8.98E-25	DEAR2; ethylene-responsive transcription factor ERF010	ethylene-responsive transcription factor ERF011
35	105033442	1.9849	Up	4.51E-24	RAP2.9; ethylene-responsive transcription factor RAP2-9; K09286 EREBP-like factor	ethylene-responsive transcription factor ERF011-like
36	105061024	1.8436	Up	8.00E-12	ERF3; ethylene-responsive transcription factor 3; K09286 EREBP-like factor	ethylene-responsive transcription factor 3-like%2C transcript variant X2
37	105046008	1.7938	Up	2.25E-11	CRF4; ethylene-responsive transcription factor CRF4	ethylene-responsive transcription factor ERF069-like
38	105047721	1.7818	Up	1.49E-09	DEAR2; ethylene-responsive transcription factor ERF010	ethylene-responsive transcription factor ERF011-like
39	105051098	1.7179	Up	0.0067838	ethylene-responsive transcription factor ERF114	ethylene-responsive transcription factor ERF113%2C transcript variant X2
40	105057222	1.717	Up	6.29E-14	ERF9; ethylene-responsive transcription factor 9; K09286 EREBP-like factor	ethylene-responsive transcription factor ERF069-like
41	105051977	1.5838	Up	0.013911	ERF110; ethylene-responsive transcription factor ERF110	ethylene-responsive transcription factor ABR1
42	105045901	1.5472	Up	5.29E-11	DREB2C; dehydration-responsive element-binding protein 2C	dehydration-responsive element-binding protein 2B-like%2C transcript variant X1
43	105048703	1.4659	Up	0.0004919	ethylene-responsive transcription factor ERF012	ethylene-responsive transcription factor ERF014-like
44	105053951	1.4264	Up	0.0004794	ERF-1; ethylene-responsive transcription factor 1A; K09286 EREBP-like factor	ethylene-responsive transcription factor 2-like
45	105055801	1.215	Up	0.0081281	dehydration-responsive element-binding protein 2G	dehydration-responsive element-binding protein 2C-like
46	105042814	1.2139	Up	5.49E-05	ethylene-responsive transcription factor ERF053	ethylene-responsive transcription factor RAP2-4-like
47	105033440	1.1478	Up	1.05E-07	AP2; Floral homeotic protein APETALA 2; K09284 AP2-like factor, euAP2 lineage	floral homeotic protein APETALA 2-like
48	105041629	1.0555	Up	0.0016823	RAP2.4; ethylene-responsive transcription factor RAP2-4; K09286 EREBP-like factor	ethylene-responsive transcription factor RAP2-4-like
49	105052809	1.0279	Up	0.02819	DEAR2; ethylene-responsive transcription factor ERF010	ethylene-responsive transcription factor ERF011-like%2C transcript variant X3
50	105047575	0.98259	Up	0.0042653	ethylene-responsive transcription factor ERF061	ethylene-responsive transcription factor ERF061-like
51	105057588	0.94121	Up	0.035121	AP2-like ethylene-responsive transcription factor; K09285 AP2-like factor, ANT lineage	AP2-like ethylene-responsive transcription factor At2g41710%2C transcript variant X2
52	105056862	0.90192	Up	4.26E-05	RAP2.4; ethylene-responsive transcription factor RAP2-4; K09286 EREBP-like factor	ethylene-responsive transcription factor RAP2-13
53	105039100	0.85196	Up	0.017353	ERF8; ethylene-responsive transcription factor 8	--
54	105038860	0.61535	Up	0.025559	Ethylene-responsive transcription factor-like protein	ethylene-responsive transcription factor-like protein At4g13040%2C transcript variant X3
55	105058589	0.43155	Up	0.041889	RAP2.12; ethylene-responsive transcription factor RAP2-12; K09286 EREBP-like factor	ethylene-responsive transcription factor 1-like%2C transcript variant X2
56	105034164	-0.82169	Down	1.69E-05	RAP2.7; TARGET OF EARLY ACTIVATION TAGGED 1; K09284 AP2-like factor, euAP2 lineage	AP2-like ethylene-responsive transcription factor TOE3%2C transcript variant X2
57	105045791	-0.83317	Down	0.0003598	RAP2.7; TARGET OF EARLY ACTIVATION TAGGED 1; K09284 AP2-like factor, euAP2 lineage	floral homeotic protein APETALA 2-like
58	105056677	-0.86828	Down	5.27E-06	RAP2.2; ethylene-responsive transcription factor RAP2-2; K09286 EREBP-like factor	ethylene-responsive transcription factor 1-like
59	105045012	-0.91372	Down	1.93E-06	ethylene-responsive transcription factor ERF118	ethylene-responsive transcription factor ERF073-like%2C transcript variant X2
60	105052807	-1.0813	Down	0.0040012	AP2; Floral homeotic protein APETALA 2; K09284 AP2-like factor, euAP2 lineage	AP2-like ethylene-responsive transcription factor TOE3%2C transcript variant X5
61	105049141	-1.1171	Down	0.0029912	AP2-like ethylene-responsive transcription factor; K09285 AP2-like factor, ANT lineage	AP2-like ethylene-responsive transcription factor At2g41710%2C transcript variant X2
62	105044670	-1.1262	Down	6.75E-10	RAP2.7; TARGET OF EARLY ACTIVATION TAGGED 1; K09284 AP2-like factor, euAP2 lineage	ethylene-responsive transcription factor RAP2-7-like%2C transcript variant X2
63	105038034	-1.1698	Down	0.0004145	ERF110; ethylene-responsive transcription factor ERF110	ethylene-responsive transcription factor ABR1-like
64	105053042	-1.3488	Down	3.79E-09	RAP2.2; ethylene-responsive transcription factor RAP2-2; K09286 EREBP-like factor	ethylene-responsive transcription factor 1-like
65	105041196	-1.4553	Down	3.75E-06	CRF2; ethylene-responsive transcription factor CRF2	ethylene-responsive transcription factor ERF073-like
66	105059101	-1.9681	Down	0.0005895	ERF1; ethylene-responsive transcription factor 1B; K14516 ethylene-responsive transcription factor 1	ethylene-responsive transcription factor 1B-like
67	105043078	-1.9797	Down	1.17E-06	ERF1; ethylene-responsive transcription factor 1B; K14516 ethylene-responsive transcription factor 1	ethylene-responsive transcription factor 1B-like
68	105040550	-2.2039	Down	0.0003754	TINY2; dehydration-responsive element-binding protein 3; K09286 EREBP-like factor	dehydration-responsive element-binding protein 3-like
69	105043684	-2.4177	Down	2.64E-05	ethylene-responsive transcription factor ERF034	ethylene-responsive transcription factor ERF038
70	105053975	-2.4807	Down	0.0026986	ERF38; ethylene-responsive transcription factor ERF038	ethylene-responsive transcription factor ERF038-like
71	105060231	-2.823	Down	0.0001487	ethylene-responsive transcription factor ERF003	ethylene-responsive transcription factor ERF003-like
72	105039661	-2.9173	Down	0.0034565	ethylene-responsive transcription factor ERF003	ethylene-responsive transcription factor ERF003-like
73	105050808	-4.9427	Down	0.002389	ethylene-responsive transcription factor ERF003	ethylene-responsive transcription factor ERF003-like

DEGs with P adjusted (Padj, P value after normalization) value smaller than 0.05 are shortlisted. The expression level is displayed in log2 fold change (drought versus control). Up-regulated DEGs are highlighted in red and down-regulated DEGs are highlighted in green. KEGG annotation information and description of genes are added.

Table S2 Expression data of differential expressed genes (DEGs) encode for bHLH transcription factors.

No	Gene Locus ID	log2FoldChange (Dro vs Ctrl)	Up-Down expression	padj	KEGG Annot Info	Description
1	105060915	3.868	Up	3.33E-08	HEC1; transcription factor HEC1	--
2	105058234	3.3752	Up	4.15E-09	HEC1; transcription factor HEC1	--
3	105050852	3.2054	Up	1.92E-49	transcription factor bHLH35	--
4	105046552	2.8765	Up	1.37E-28	BHLH92; transcription factor bHLH92	ABSCISIC ACID-INSENSITIVE 5-like protein 2
5	105056299	2.5761	Up	8.88E-28	basic helix-loop-helix domain-containing protein	--
6	105059673	2.5371	Up	0.00744	transcription factor bHLH61	--
7	105048079	2.53	Up	2.16E-10	basic helix-loop-helix domain-containing protein	uncharacterized LOC105055101|
8	105043646	2.047	Up	1.43E-23	UNE10; transcription factor UNE10	basic leucine zipper 61-like
9	105033687	1.9943	Up	0.00024	TT8; transcription factor TT8	ocs element-binding factor 1-like
10	105053039	1.7775	Up	9.84E-15	LRL3; bHLH protein LJRHL1-like 3	--
11	105053846	1.5036	Up	3.90E-16	MYC2; transcription factor MYC2; K13422 transcription factor MYC2	--
12	105047972	1.4612	Up	2.10E-08	basic helix-loop-helix domain-containing protein	--
13	105055398	1.2872	Up	1.24E-07	ILR3; transcription factor ILR3	--
14	105058928	1.1248	Up	1.31E-08	ILR3; transcription factor ILR3	--
15	105041228	1.1198	Up	0.010199	BEE1; transcription factor BEE 1	basic leucine zipper 61-like%2C transcript variant X2
16	105059368	1.1059	Up	0.006748	MYC2; transcription factor MYC2; K13422 transcription factor MYC2	--
17	105049326	1.0761	Up	0.002707	transcription factor bHLH35	uncharacterized LOC105057834
18	105047615	1.0004	Up	1.81E-05	transcription factor bHLH13	ocs element-binding factor 1-like
19	105055031	0.96702	Up	0.000127	MYC2; transcription factor MYC2; K13422 transcription factor MYC2	--
20	105058460	0.92768	Up	0.002882	transcription factor bHLH96	--
21	105038134	0.70646	Up	0.001025	transcription factor bHLH13	transcription factor HY5-like
22	105055797	0.5726	Up	0.022408	UNE12; transcription factor UNE12	--
23	105058719	0.57141	Up	0.010515	basic helix-loop-helix domain-containing protein	--
24	105040114	0.56517	Up	0.006802	PIF3; transcription factor PIF3; K12126 phytochrome-interacting factor 3	DNA-binding protein EMBP-1-like%2C transcript variant X2
25	105035562	-0.62221	Down	0.004677	PIF3; transcription factor PIF3; K12126 phytochrome-interacting factor 3	G-box-binding factor 3-like%2C transcript variant X1
26	105040678	-0.65094	Down	0.0326	transcription factor bHLH30	ocs element-binding factor 1-like
27	105050232	-0.76358	Down	9.25E-05	transcription factor bHLH130	transcription factor VIP1-like
28	105044567	-0.81705	Down	0.048944	transcription factor bHLH51	ABSCISIC ACID-INSENSITIVE 5-like protein 7%2C transcript variant X3
29	105059744	-0.89754	Down	7.53E-06	PIF3; transcription factor PIF3; K12126 phytochrome-interacting factor 3	--
30	105032028	-1.0809	Down	7.78E-09	PIL5; transcription factor PIF1	transcription factor HBP-1b(c38)-like%2C transcript variant X1
31	105033626	-1.0922	Down	5.72E-06	PIL6; transcription factor PIF5	transcription factor TGA2-like
32	105050500	-1.0925	Down	0.047919	transcription factor bHLH60	bZIP transcription factor TRAB1%2C transcript variant X1
33	105050721	-1.1711	Down	0.002842	LRL1; bHLH protein LJRHL1-like 1	G-box-binding factor 1-like%2C transcript variant X1
34	105049473	-1.3099	Down	0.002449	SPT; transcription factor SPATULA	bZIP transcription factor TRAB1%2C transcript variant X3
35	105058167	-1.346	Down	0.000626	transcription factor bHLH25	--
36	105048892	-1.646	Down	9.08E-15	transcription factor bHLH62	transcription factor RF2b-like%2C transcript variant X2
37	105052814	-1.6484	Down	1.66E-07	SPT; transcription factor SPATULA	--
38	105032410	-1.6616	Down	1.85E-05	transcription factor bHLH62	protein ABSCISIC ACID-INSENSITIVE 5-like%2C transcript variant X2
39	105043257	-1.8855	Down	3.50E-10	transcription factor bHLH67	G-box-binding factor 3-like%2C transcript variant X2
40	105060198	-1.893	Down	8.05E-23	LRL1; bHLH protein LJRHL1-like 1	--
41	105056257	-1.9914	Down	0.014239	CIB1; cryptochrome-interacting basic-helix-loop-helix 1	--
42	105038881	-2.0294	Down	0.001656	transcription factor bHLH62	transcription factor HY5
43	105058341	-2.0961	Down	0.012633	KDR; basic helix-loop-helix protein KIDARI	--
44	105055150	-2.2109	Down	0.001242	transcription factor bHLH61	--
45	105048427	-2.8143	Down	0.000395	transcription factor CESTA	ocs element-binding factor 1-like
46	105049757	-2.9218	Down	0.000467	transcription factor bHLH137	transcription factor HBP-1a-like%2C transcript variant X2
47	105033952	-3.2356	Down	1.88E-11	transcription factor bHLH62	transcription factor TGA2-like%2C transcript variant X3
48	105040557	-4.4828	Down	1.92E-05	basic helix-loop-helix domain-containing protein	probable transcription factor PosF21%2C transcript variant X1
49	105035052	-6.6871	Down	0.00371	transcription factor bHLH137	uncharacterized protein At4g06598-like

DEGs with P adjusted (Padj, P value after normalization) value smaller than 0.05 are shortlisted. The expression level is displayed in log2 fold change (drought versus control). Up-regulated DEGs are highlighted in red and down-regulated DEGs are highlighted in green. KEGG annotation information and description of genes are added.

Table S3 Expression data of differential expressed genes (DEGs) encode for bZIP transcription factors.

No	Gene Locus ID	log2FoldChange (Dro vs Ctrl)	Up-Down expression	padj	KEGG Annot Info	Description
1	105047023	3.2121	Up	5.58E-33	GBF6; G-box binding factor 6	ocs element-binding factor 1-like
2	105040116	2.7114	Up	5.40E-29	HYH; transcription factor HY5-like protein	transcription factor HY5-like
3	105059360	2.0304	Up	7.97E-20	Basic-leucine zipper (bZIP) transcription factor family protein	transcription factor VIP1-like
4	105058236	1.9426	Up	1.09E-24	ABF4; ABRE binding factor 4; K14432 ABA responsive element binding factor	bZIP transcription factor TRAB1%2C transcript variant X3
5	105053767	1.7977	Up	2.17E-12	bZIP23; transcription factor bZIP23	--
6	105057202	1.7273	Up	0.000253	bZIP transcription factor	transcription factor RF2b-like%2C transcript variant X2
7	105060907	1.5044	Up	2.60E-15	ABF2; abscisic acid responsive elements-binding factor 2; K14432 ABA responsive element binding factor	bZIP transcription factor TRAB1%2C transcript variant X1
8	105057834	1.3978	Up	5.41E-13	BZIP17; transcription factor BZIP17	uncharacterized LOC105057834
9	105046532	1.3642	Up	0.001233	GBF1; G-box binding factor 1; K09060 plant G-box-binding factor	DNA-binding protein EMBP-1-like%2C transcript variant X2
10	105052591	1.196	Up	6.20E-10	BZIP53; basic region/leucine zipper motif 53 protein	ocs element-binding factor 1-like
11	105039703	1.1873	Up	1.80E-09	GBF3; G-box binding factor 3; K09060 plant G-box-binding factor	G-box-binding factor 3-like%2C transcript variant X1
12	105046738	1.1827	Up	1.59E-08	Basic-leucine zipper (bZIP) transcription factor family protein	probable transcription factor PosF21%2C transcript variant X1
13	105040182	1.1059	Up	2.67E-07	HY5; transcription factor HY5; K16241 transcription factor HY5	transcription factor HY5
14	105038245	0.83148	Up	0.034554	bZIP transcription factor family protein; K14431 transcription factor TGA	transcription factor TGA2-like%2C transcript variant X3
15	105037133	0.8307	Up	0.004174	BZIP53; basic region/leucine zipper motif 53 protein	ocs element-binding factor 1-like
16	105047544	0.80579	Up	0.000228	GBF3; G-box binding factor 3; K09060 plant G-box-binding factor	G-box-binding factor 3-like%2C transcript variant X2
17	105060966	0.71199	Up	0.02624	GBF1; G-box binding factor 1; K09060 plant G-box-binding factor	G-box-binding factor 1-like%2C transcript variant X1
18	105055101	0.67422	Up	0.001526	bZIP19; transcription factor bZIP19	uncharacterized LOC105055101
19	105049450	0.59795	Up	0.012603	GBF4; G-box binding factor 4; K14432 ABA responsive element binding factor	ABSCISIC ACID-INSENSITIVE 5-like protein 7%2C transcript variant X3
20	105032048	-0.47214	Down	0.031686	AHBP-1B; transcription factor TGA2; K14431 transcription factor TGA	transcription factor HBP-1b(c38)-like%2C transcript variant X1
21	105038510	-0.64094	Down	0.001621	bZIP transcription factor	uncharacterized protein At4g06598-like
22	105058685	-0.70692	Down	0.010483	bZIP16; basic region/leucine zipper transcription factor 16; K09060 plant G-box-binding factor	transcription factor HBP-1a-like%2C transcript variant X2
23	105034229	-1.6351	Down	0.015107	bZIP transcription factor family protein; K14431 transcription factor TGA	transcription factor TGA2-like
24	105052394	-1.9778	Down	9.31E-20	AREB3; ABA-responsive element binding protein 3; K14432 ABA responsive element binding factor	ABSCISIC ACID-INSENSITIVE 5-like protein 2
25	105034225	-2.2189	Down	6.25E-11	ABI5; protein abscisic acid-insensitive 5; K14432 ABA responsive element binding factor	protein ABSCISIC ACID-INSENSITIVE 5-like%2C transcript variant X2
26	105047271	-2.4868	Down	0.000727	BZIP61; transcription factor BZIP61	basic leucine zipper 61-like%2C transcript variant X2
27	105048165	-3.7058	Down	5.67E-05	BZIP34; transcription factor BZIP34	basic leucine zipper 61-like
28	105056236	-3.8028	Down	3.30E-83	GBF6; G-box binding factor 6	ocs element-binding factor 1-like

DEGs with P adjusted (Padj, P value after normalization) value smaller than 0.05 are shortlisted. The expression level is displayed in log2 fold change (drought versus control). Up-regulated DEGs are highlighted in red and down-regulated DEGs are highlighted in green. KEGG annotation information and description of genes are added.

Table S4 Expression data of differential expressed genes (DEGs) encode for C2H2 transcription factors.

No	Gene Locus ID	log2FoldChange (Dro vs Ctrl)	Up-Down expression	padj	KEGG Annot Info	Description
1	105036887	5.0278	Up	3.25E-40	C2H2-type zinc finger-containing protein	zinc finger protein ZAT8-like
2	105032835	4.1083	Up	3.18E-53	zinc finger protein ZAT11	zinc finger protein ZAT11-like
3	105044475	3.696	Up	1.37E-25	C2H2-type zinc finger-containing protein	zinc finger protein ZAT11-like
4	105060714	3.5326	Up	7.53E-36	STZ; zinc finger protein STZ/ZAT10	zinc finger protein ZAT10-like
5	105045719	3.3074	Up	0.001894	zinc finger protein ZAT11	zinc finger protein ZAT11
6	105033259	2.6812	Up	5.35E-17	STZ; zinc finger protein STZ/ZAT10	zinc finger protein 1-like
7	105034068	2.5297	Up	2.14E-15	STZ; zinc finger protein STZ/ZAT10	zinc finger protein ZAT10-like
8	105051874	2.2624	Up	1.45E-28	STOP1; protein SENSITIVE TO PROTON RHIZOTOXICITY 1	zinc finger protein STOP1 homolog||zinc finger protein STOP1 homolog
9	105045723	2.03	Up	0.005146	C2H2-type zinc finger protein	zinc finger protein ZAT11-like
10	105044471	2.0278	Up	0.026784	C2H2-type zinc finger-containing protein	zinc finger protein ZAT12-like
11	105032342	1.8842	Up	3.41E-09	C2H2-like zinc finger protein	zinc finger protein NUTCRACKER-like
12	105035784	1.8668	Up	5.81E-23	STOP1; protein SENSITIVE TO PROTON RHIZOTOXICITY 1	zinc finger protein STOP1 homolog%2C transcript variant X1
13	105032838	1.5535	Up	1.36E-05	zinc finger protein ZAT5	zinc finger protein ZAT12-like
14	105038870	1.5505	Up	2.77E-05	WIP3; WIP domain protein 3	zinc finger protein WIP3-like
15	105047798	0.64336	Up	0.003545	C2H2-like zinc finger protein	uncharacterized LOC105047798%2C transcript variant X10
16	105058080	0.55155	Up	0.027926	DNAJ heat shock N-terminal domain-containing protein; K09506	dnaJ homolog subfamily C member 21
17	105039701	-0.4807	Down	0.033205	SUF4; protein SUPPRESSOR of FRI 4	protein SUPPRESSOR OF FRI 4-like
18	105056808	-0.51075	Down	0.044245	MBD8; methyl-CPG-binding domain-containing protein	uncharacterized LOC105056808
19	105035044	-0.54513	Down	0.010763	HDA3; histone deacetylase HDT1	histone deacetylase HDT2-like
20	105045823	-0.54797	Down	0.035875	C2H2-like zinc finger protein	zinc finger protein JACKDAW-like
21	105031955	-0.60229	Down	0.0022	IDD5; indeterminate(ID)-domain 5 protein	zinc finger protein NUTCRACKER-like%2C transcript variant X1
22	105035474	-0.6186	Down	0.009743	HDA3; histone deacetylase HDT1	histone deacetylase HDT2-like%2C transcript variant X3
23	105045430	-0.72826	Down	0.006537	TFIIIA; transcription factor IIIA; K09191 general transcription fac	zinc finger protein 91-like
24	105054000	-0.77167	Down	0.049897	C2H2-like zinc finger protein	zinc finger protein ZAT9-like
25	105035330	-0.81996	Down	0.001512	C2H2-like zinc finger protein	--
26	105036747	-0.86405	Down	0.000448	C2H2-type zinc finger protein	zinc finger protein ZAT5-like
27	105055287	-0.94956	Down	4.29E-07	IDD5; indeterminate(ID)-domain 5 protein	zinc finger protein NUTCRACKER-like%2C transcript variant X4
28	105055625	-1.1985	Down	0.00144	C2H2-type zinc finger protein	zinc finger protein ZAT5-like
29	105058669	-1.2452	Down	0.00982	IDD16; shoot gravitropism5-like protein	protein SHOOT GRAVITROPISM 5-like
30	105060971	-1.2643	Down	5.35E-12	C2H2-like zinc finger protein	zinc finger protein MAGPIE-like%2C transcript variant X1
31	105041918	-1.7511	Down	6.37E-05	SGR5; protein shoot gravitropism 5	protein SHOOT GRAVITROPISM 5-like
32	105056371	-1.9361	Down	0.046791	IDD16; shoot gravitropism5-like protein	protein SHOOT GRAVITROPISM 5-like
33	105055656	-2.115	Down	0.020181	zinc finger-like protein	protein SENSITIVE TO PROTON RHIZOTOXICITY 1-like

DEGs with P adjusted (Padj, P value after normalization) value smaller than 0.05 are shortlisted. The expression level is displayed in log2 fold change (drought versus control). Up-regulated DEGs are highlighted in red and down-regulated DEGs are highlighted in green. KEGG annotation information and description of genes are added.

Table S5 Expression data of differential expressed genes (DEGs) encode for MYB transcription factors.

No	Gene Locus ID	log2FoldChange (Dro vs Ctrl)	Up-Down expression	padj	KEGG Annot Info	Description
1	105056444	4.37	Up	1.55E-14	MYB5; transcription repressor MYB5; K09422 myb proto-oncogene protein, plant	anthocyanin regulatory C1 protein-like
2	105044973	4.2687	Up	0.002575	MYB108; putative transcription factor MYB108; K09422 myb proto-oncogene protein, plant	transcription factor MYB108
3	105056442	4.1588	Up	1.56E-55	MYB12; transcription factor MYB12; K09422 myb proto-oncogene protein, plant	myb-related protein Myb4-like
4	105056443	4.0272	Up	1.88E-46	MYB5; transcription repressor MYB5; K09422 myb proto-oncogene protein, plant	anthocyanin regulatory C1 protein-like
5	105033428	3.5924	Up	3.48E-08	MYB14; myb domain protein 14; K09422 myb proto-oncogene protein, plant	myb-related protein Myb4-like
6	105032141	3.2948	Up	0.000373	MYB102; R2R3 family MYB transcription factor; K09422 myb proto-oncogene protein, plant	transcription factor MYB3-like
7	105048449	3.1001	Up	0.000856	MYB4; transcription repressor MYB4; K09422 myb proto-oncogene protein, plant	transcription repressor MYB4-like
8	105046865	2.7573	Up	5.88E-39	CPC; transcription factor CPC; K09422 myb proto-oncogene protein, plant	transcription factor CPC-like
9	105057307	2.5681	Up	0.000596	MYB108; putative transcription factor MYB108; K09422 myb proto-oncogene protein, plant	transcription factor MYB108-like
10	105061441	2.5517	Up	1.80E-08	duplicated SANT DNA-binding domain-containing protein	transcription factor DIVARICATA-like%2C transcript variant X1
11	105033230	2.468	Up	7.83E-27	TT2; transcription factor TT2; K09422 myb proto-oncogene protein, plant	transcription repressor MYB5-like
12	105056584	2.3016	Up	3.97E-05	MYB94; putative transcription factor MYB94; K09422 myb proto-oncogene protein, plant	myb-related protein 306-like
13	105048425	2.1141	Up	2.48E-06	MYB62; R2R3-MYB transcription family; K09422 myb proto-oncogene protein, plant	myb-related protein 305-like%2C transcript variant X1
14	105043705	2.1032	Up	2.20E-12	DUO1; R2R3 myb transcription factor DUO1; K09422 myb proto-oncogene protein, plant	transcription factor MYB48
15	105053103	1.9279	Up	0.022877	TT2; transcription factor TT2; K09422 myb proto-oncogene protein, plant	transcription factor TT2-like
16	105050171	1.8918	Up	1.66E-24	MYBR1; transcription factor MYB44; K09422 myb proto-oncogene protein, plant	transcription factor MYB44-like
17	105058870	1.5666	Up	2.09E-13	RVE2; MYB family transcription factor Circadian 1	protein CCA1-like
18	105048057	1.5597	Up	1.19E-08	MYB5; transcription repressor MYB5; K09422 myb proto-oncogene protein, plant	transcription repressor MYB5-like
19	105041192	1.4718	Up	1.02E-07	MYB4; transcription repressor MYB4; K09422 myb proto-oncogene protein, plant	myb-related protein 308-like
20	105045660	1.3952	Up	1.34E-07	AS1; transcription factor AS1; K09422 myb proto-oncogene protein, plant	transcription factor AS1-like%2C transcript variant X2
21	105058320	1.3331	Up	0.002326	MYB94; putative transcription factor MYB94; K09422 myb proto-oncogene protein, plant	myb-related protein 306
22	105060999	1.2872	Up	4.97E-12	MYB73; myb domain protein 73; K09422 myb proto-oncogene protein, plant	transcription factor MYB44-like
23	105054972	1.2177	Up	2.49E-09	myb family transcription factor	protein REVEILLE 8-like
24	105054751	1.2174	Up	8.82E-09	myb-like transcription factor family protein	uncharacterized LOC105054751%2C transcript variant X3
25	105053102	1.2071	Up	3.05E-06	MYB5; transcription repressor MYB5; K09422 myb proto-oncogene protein, plant	transcription factor WER-like%2C transcript variant X2
26	105061460	1.1848	Up	7.90E-05	duplicated SANT DNA-binding domain-containing protein	transcription factor DIVARICATA-like
27	105043254	1.1684	Up	4.17E-10	CCA1; protein CCA1; K12134 circadian clock associated 1	protein LHY-like%2C transcript variant X2
28	105042228	1.1436	Up	0.036363	MYB43; myb domain protein 43; K09422 myb proto-oncogene protein, plant	protein ODORANT1-like
29	105040532	1.1214	Up	0.009971	MYB5; transcription repressor MYB5; K09422 myb proto-oncogene protein, plant	uncharacterized LOC105040532
30	105058636	1.0785	Up	4.46E-06	TT2; transcription factor TT2; K09422 myb proto-oncogene protein, plant	transcription factor TT2-like
31	105051250	1.0428	Up	0.004844	CPC; transcription factor CPC; K09422 myb proto-oncogene protein, plant	transcription factor CPC-like
32	105032833	1.0288	Up	0.046324	MYB59; transcription factor MYB59; K09422 myb proto-oncogene protein, plant	myb-related protein MYBAS2-like%2C transcript variant X1
33	105048799	0.87952	Up	0.005065	MYB94; putative transcription factor MYB94; K09422 myb proto-oncogene protein, plant	myb-related protein 306-like
34	105052936	0.82605	Up	0.021499	MYBR1; transcription factor MYB44; K09422 myb proto-oncogene protein, plant	transcription factor MYB44-like
35	105041706	0.78419	Up	0.021535	FLP; MYB transcription factor FLP; K09422 myb proto-oncogene protein, plant	transcription factor MYB76%2C transcript variant X2
36	105055259	0.76037	Up	0.000157	RVE2; MYB family transcription factor Circadian 1	protein CCA1%2C transcript variant X2
37	105041730	0.74729	Up	0.01812	MYB94; putative transcription factor MYB94; K09422 myb proto-oncogene protein, plant	myb-related protein 306-like
38	105036987	0.57288	Up	0.039596	myb-like transcription factor family protein	transcription factor MYB1R1-like
39	105055455	0.54928	Up	0.009969	duplicated SANT DNA-binding domain-containing protein	transcription factor DIVARICATA%2C transcript variant X1
40	105050915	0.47794	Up	0.036799	myb-like HTH transcriptional regulator family protein	uncharacterized LOC105050915
41	105054760	0.44293	Up	0.049971	myb-like transcription factor family protein	transcription factor MYB1R1-like
42	105059464	0.4353	Up	0.049802	myb-like transcription factor family protein	uncharacterized LOC105059464%2C transcript variant X2
43	105046273	-0.58504	Down	0.010622	Duplicated homeodomain-like superfamily protein	transcription factor DIVARICATA-like
44	105056239	-0.62712	Down	0.003651	DNA binding / transcription factor; K15198 transcription factor TFIIIB component B''	uncharacterized LOC105056239%2C transcript variant X4
45	105036764	-0.63382	Down	0.004845	MYB4; transcription repressor MYB4; K09422 myb proto-oncogene protein, plant	myb-related protein 308-like
46	105059291	-0.67742	Down	0.009197	DNA binding / transcription factor; K15198 transcription factor TFIIIB component B''	uncharacterized LOC105059291%2C transcript variant X2
47	105045069	-0.71495	Down	0.037811	MYB111; myb domain protein 111; K09422 myb proto-oncogene protein, plant	myb-related protein P-like
48	105039452	-0.85862	Down	2.60E-05	DNA-binding bromodomain-containing protein	uncharacterized LOC105039452%2C transcript variant X2
49	105048294	-1.0385	Down	0.000172	PC-MYB1; myb-related protein 3R-1	myb-related protein 3R-1-like%2C transcript variant X2
50	105034252	-1.1336	Down	3.47E-07	Duplicated homeodomain-like superfamily protein	transcription factor DIVARICATA-like
51	105056637	-1.1742	Down	6.75E-07	MYB106; myb domain protein 106; K09422 myb proto-oncogene protein, plant	myb-related protein Pp2-like
52	105046084	-1.3068	Down	0.046436	MYB117; myb-domain transcription factor LOF1; K09422 myb proto-oncogene protein, plant	transcription factor MYB122-like
53	105055088	-1.3494	Down	1.85E-06	MYB4; transcription repressor MYB4; K09422 myb proto-oncogene protein, plant	myb-related protein Hv1-like
54	105054576	-1.4351	Down	0.003839	MYB85; myb domain protein 85; K09422 myb proto-oncogene protein, plant	protein ODORANT1-like
55	105052376	-1.4586	Down	0.016196	MYB60; putative transcription factor MYB60; K09422 myb proto-oncogene protein, plant	myb-related protein 306-like%2C transcript variant X1
56	105037388	-1.5312	Down	0.030627	MYB63; myb domain protein 63; K09422 myb proto-oncogene protein, plant	myb-related protein Zm1-like
57	105035724	-1.6615	Down	0.005973	MYB84; transcription factor RAX3; K09422 myb proto-oncogene protein, plant	transcription factor RAX2-like
58	105057270	-1.7947	Down	4.64E-06	MYB105; MYB-domain transcription factor LOF2; K09422 myb proto-oncogene protein, plant	myb-related protein MYBAS2-like%2C transcript variant X5
59	105042961	-1.8949	Down	0.00149	MYB36; putative transcription factor MYB36; K09422 myb proto-oncogene protein, plant	transcription factor RAX2-like
60	105032366	-2.1974	Down	0.033754	MYB17; LATE MERISTEM IDENTITY2; K09422 myb proto-oncogene protein, plant	--
61	105046138	-2.265401671	Down	2.06E-02	--	inositol phosphorylceramide glucuronosyltransferase 1
62	105034382	-2.4225	Down	0.015107	CPL3; CAPRICE-like MYB3; K09422 myb proto-oncogene protein, plant	uncharacterized LOC105034382%2C transcript variant X2
63	105053200	-2.5504	Down	0.023814	MYB43; myb domain protein 43; K09422 myb proto-oncogene protein, plant	protein ODORANT1-like

DEGs with P adjusted (Padj, P value after normalization) value smaller than 0.05 are shortlisted. The expression level is displayed in log2 fold change (drought versus control). Up-regulated DEGs are highlighted in red and down-regulated DEGs are highlighted in green. KEGG annotation information and description of genes are added.

Table S6 Expression data of differential expressed genes (DEGs) encode for NAC transcription factors.

No	Gene Locus ID	log2FoldChange (Dro vs Ctrl)	Up-Down expression	padj	KEGG Annot Info	Description
1	105057259	6.7203	Up	1.30E-93	NAC032; NAC domain containing protein 32	NAC domain-containing protein 68-like
2	105042635	4.9678	Up	3.84E-93	NAC036; NAC transcription factor family protein NAC036	putative NAC domain-containing protein 94
3	105061462	4.2938	Up	3.45E-51	NAC090; NAC domain-containing protein	NAC domain-containing protein 90-like
4	105044900	3.4901	Up	8.83E-47	ATAF2; protein ATAF2	NAC domain-containing protein 68-like
5	105047002	3.4529	Up	0.00093733	NAC2; NAC domain containing protein 2	NAC transcription factor 25-like
6	105055179	3.3417	Up	9.88E-05	NAC036; NAC transcription factor family protein NAC036	protein FEZ-like
7	105057261	3.0509	Up	1.66E-06	NAC047; NAC domain containing protein 47	NAC transcription factor 29-like
8	105037120	2.9947	Up	1.89E-42	ATAF1; putative transcriptional activator with NAC domain	NAC domain-containing protein 48-like
9	105038428	2.8253	Up	4.05E-09	ATAF1; putative transcriptional activator with NAC domain	NAC domain-containing protein 48-like%2C transcript variant X2
10	105061547	2.8134	Up	1.12E-08	NAC036; NAC transcription factor family protein NAC036	putative NAC domain-containing protein 94
11	105036710	2.6599	Up	1.24E-22	ATAF1; putative transcriptional activator with NAC domain	NAC domain-containing protein 48-like
12	105033559	2.5662	Up	5.60E-28	NAC1; transcription factor NAC1	NAC domain-containing protein 21/22-like
13	105044906	2.468	Up	2.06E-09	NAC100; NAC domain containing protein 100	NAC domain-containing protein 100-like%2C transcript variant X1
14	105032026	2.29	Up	6.61E-20	NAC1; transcription factor NAC1	NAC domain-containing protein 21/22-like
15	105046073	2.2506	Up	1.26E-05	NAC047; NAC domain containing protein 47	NAC transcription factor 29-like
16	105051813	2.2085	Up	1.06E-32	ATAF1; putative transcriptional activator with NAC domain	NAC domain-containing protein 48-like
17	105056230	2.046	Up	2.18E-28	NAC028; NAC domain containing protein 28	NAC domain-containing protein 74-like%2C transcript variant X1
18	105047003	1.8164	Up	1.15E-13	NAC3; ATAF-like NAC-domain transcription factor	NAC domain-containing protein 72-like
19	105057977	1.3585	Up	1.42E-09	NAC028; NAC domain containing protein 28	NAC domain-containing protein 12-like%2C transcript variant X3
20	105052358	1.2746	Up	8.82E-09	NAC102; NAC domain-containing protein 102	NAC domain-containing protein 68-like%2C transcript variant X1
21	105060954	0.82608	Up	0.0069622	NAC057; NAC domain containing protein 57	NAC domain-containing protein 78-like%2C transcript variant X2
22	105059551	0.5839	Up	0.0061036	NAC014; protein NAC 014	uncharacterized LOC105059551%2C transcript variant X2
23	105046069	0.5191	Up	0.015377	NAC032; NAC domain containing protein 32	NAC domain-containing protein 68-like%2C transcript variant X1
24	105037721	-0.48345	Down	0.041299	NAC2; NAC family transcriptional activator	NAC domain-containing protein 48-like%2C transcript variant X1
25	105054253	-0.65287	Down	0.0034196	NTL9; NAC transcription factor-like 9	uncharacterized LOC105054253%2C transcript variant X1
26	105034190	-0.76412	Down	0.0050631	NAC035; protein LONG VEGETATIVE PHASE 1	transcription factor JUNGBRUNNEN 1
27	105034281	-1.1535	Down	9.24E-08	NAC083; NAC domain containing protein 83	NAC transcription factor 25-like
28	105051041	-1.1923	Down	1.28E-10	NAC083; NAC domain containing protein 83	NAC transcription factor 25-like
29	105054488	-1.2737	Down	0.036725	NAC1; transcription factor NAC1	NAC domain-containing protein 21/22-like
30	105061407	-1.3693	Down	3.85E-08	NAC083; NAC domain containing protein 83	NAC transcription factor 25-like
31	105058562	-1.4791	Down	3.31E-09	NAP; NAC transcription factor protein family	NAC transcription factor 29-like%2C transcript variant X2
32	105059815	-1.6149	Down	0.009608	NAC1; transcription factor NAC1	NAC domain-containing protein 21/22-like
33	105038579	-3.2019	Down	1.90E-46	NAC035; protein LONG VEGETATIVE PHASE 1	protein ATAF2-like

DEGs with P adjusted (Padj, P value after normalization) value smaller than 0.05 are shortlisted. The expression level is displayed in log2 fold change (drought versus control). Up-regulated DEGs are highlighted in red and down-regulated DEGs are highlighted in green. KEGG annotation information and description of genes are added.

Table S7 Expression data of differential expressed genes (DEGs) encode for Orphans transcription factors.

No	Gene Locus ID	log2FoldChange (Dro vs Ctrl)	Up-Down expression	padj	KEGG Annot Info	Description
1	105051131	3.2559	Up	5.04E-05	zinc finger protein CONSTANS-LIKE 14	--
2	105058925	2.5604	Up	2.65E-21	ARR3; response regulator 3; K14492 two-component response regulator ARR-A family	two-component response regulator ARR3-like
3	105047331	2.481	Up	0.001416	CCT motif family protein	zinc finger protein CONSTANS-LIKE 1-like
4	105049947	2.1264	Up	2.61E-14	zinc finger protein CONSTANS-LIKE 10	zinc finger protein CONSTANS-LIKE 9-like
5	105043504	2.0055	Up	7.15E-06	putative zinc finger transcription factor BZS1	B-box zinc finger protein 20-like%2C transcript variant X2
6	105055597	1.8618	Up	3.17E-22	EIN4; protein EIN4; K14509 ethylene receptor [EC:2.7.13.-]	protein EIN4%2C transcript variant X3
7	105060284	1.5709	Up	2.77E-17	COL2; zinc finger protein CONSTANS-LIKE 2	zinc finger protein CONSTANS-LIKE 2-like
8	105045540	1.5455	Up	4.11E-07	ARR9; two-component response regulator ARR9; K14492 two-component response regulator ARR-A family	two-component response regulator ARR9-like
9	105048999	1.3053	Up	0.00078	NET superfamily protein EMB1674	uncharacterized LOC105048999
10	105048088	1.163	Up	9.94E-09	LZF1; light-regulated zinc finger protein 1	B-box zinc finger protein 22
11	105056393	0.64549	Up	0.00283	WOL; histidine kinase 4; K14489 arabidopsis histidine kinase 2/3/4 (cytokinin receptor) [EC:2.7.13.3]	histidine kinase 4-like%2C transcript variant X2
12	105058682	-0.55824	Down	0.020196	WOL; histidine kinase 4; K14489 arabidopsis histidine kinase 2/3/4 (cytokinin receptor) [EC:2.7.13.3]	histidine kinase 4-like
13	105059949	-0.59752	Down	0.015618	LIM domain-containing protein	protein DA1-related 1-like%2C transcript variant X5
14	105052987	-0.78883	Down	0.008004	CCT motif family protein	uncharacterized LOC105052987
15	105033838	-0.80873	Down	0.00189	zinc finger protein CONSTANS-LIKE 14	zinc finger protein CONSTANS-LIKE 14-like
16	105052748	-0.89028	Down	6.83E-05	ARR9; two-component response regulator ARR9; K14492 two-component response regulator ARR-A family	two-component response regulator ARR8-like
17	105038536	-0.91645	Down	0.027439	ARR3; response regulator 3; K14492 two-component response regulator ARR-A family	two-component response regulator ARR17-like%2C transcript variant X2
18	105057575	-0.99593	Down	0.030515	CCT motif family protein	uncharacterized LOC105057575
19	105048546	-1.0939	Down	0.012717	B-box type zinc finger protein with CCT domain	putative zinc finger protein CONSTANS-LIKE 11%2C transcript variant X3
20	105042336	-1.1592	Down	1.09E-05	PPD2; protein PEAPOD2	protein TIFY 4B-like%2C transcript variant X8
21	105055384	-1.253	Down	0.015176	ARR9; two-component response regulator ARR9; K14492 two-component response regulator ARR-A family	two-component response regulator ARR3-like
22	105048509	-1.8349	Down	1.37E-06	zinc finger protein CONSTANS-LIKE 16	zinc finger protein CONSTANS-LIKE 16-like
23	105049568	-1.8353	Down	0.00028	CCT motif family protein	uncharacterized LOC105049568
24	105051561	-2.1223	Down	2.23E-20	LZF1; light-regulated zinc finger protein 1	B-box zinc finger protein 22-like%2C transcript variant X1
25	105042547	-2.2963	Down	9.53E-05	CCT motif family protein	uncharacterized LOC105042547
26	105049107	-3.1621	Down	0.001447	RR3; response regulator 3; K14492 two-component response regulator ARR-A family	two-component response regulator ARR8-like

DEGs with P adjusted (Padj, P value after normalization) value smaller than 0.05 are shortlisted. The expression level is displayed in log2 fold change (drought versus control). Up-regulated DEGs are highlighted in red and down-regulated DEGs are highlighted in green. KEGG annotation information and description of genes are added.

Table S8 Expression data of differential expressed genes (DEGs) encode for SBP transcription factors.

No	Gene Locus ID	log2FoldChange (Dro vs Ctrl)	Up-Down expression	padj	KEGG Annot Info	Description
1	105038812	1.9144	Up	0.033252	squamosa promoter-binding-like protein 13	squamosa promoter-binding-like protein 16
2	105061560	1.0795	Up	2.10E-06	SPL4; squamosa promoter binding protein-like 4	squamosa promoter-binding protein 1-like
3	105042144	0.95823	Up	0.003885	squamosa promoter-binding-like protein 13	squamosa promoter-binding-like protein 18%2C transcript variant X2
4	105052876	0.89156	Up	3.53E-06	SPL14; squamosa promoter binding-like protein 14	squamosa promoter-binding-like protein 15%2C transcript variant X1
5	105049373	-0.45763	Down	0.03862	SPL14; squamosa promoter binding-like protein 14	squamosa promoter-binding-like protein 15%2C transcript variant X1
6	105061255	-0.93926	Down	0.041826	SPL9; squamosa promoter-binding-like protein 9	squamosa promoter-binding-like protein 14%2C transcript variant X3

DEGs with P adjusted (Padj, P value after normalization) value smaller than 0.05 are shortlisted. The expression level is displayed in log2 fold change (drought versus control). Up-regulated DEGs are highlighted in red and down-regulated DEGs are highlighted in green. KEGG annotation information and description of genes are added.
