# Supplementary material for: Identification of drought responsive Elaeis guineensis WRKY transcription factors with sensitivity to other abiotic stresses and hormone treatments
Source: BMC Genomics. 2022 Feb 26;23:164. doi: 10.1186/s12864-022-08378-y (PMC8882277; doi:10.1186/s12864-022-08378-y)
Supplement: Supplementary file 2 — Additional file 2: Table S9. qRT-PCR primers [file 12864_2022_8378_MOESM2_ESM.pdf]

Table S9 qRT-PCR primers

Primer name	Forward primer sequence	Reverse primer sequence
EgWRKY03	TGAAGACAGCACGACGAATAA	CACCGCCGATTTCATTCTAAAC
EgWRKY07	GGACCACAACCTGCTTCTAA	GGAAGGGAAGGAGAAGGATAATG
EgWRKY18	GTCAACAAAGCCTGGCAAAC	CCTGGCGATGTCCCAAATAA
EhWRKY26	GGAAATGGAGGATCAAGTACCC	GGAGGCGAGGAGAAGATTTG
EgWRKY27	AGTATGGCCAGAAACCCATAAA	GCG GTG TAG GTG ATG ATG AA
EgWRKY28	GATGCATGTCGTTGCTCTAATG	ACG ATC TTT CCA TCG GTC TTC
EgWRKY29	CGCCCTCCTCCTATCTCTTAT	TGTGTGTGTGTGTGTGAGAG
EgWRKY40	GAAGTATGGGCAGAAGACGATTA	CCTGTTGCACGGTCTTAGTT
EgWRKY52	GCTTCTCTCCTTCATTCGTCTC	GACTCGGCTGTTGGAAGATT
EgWRKY56	AAAGTGGATGCGGGATGTAG	GCTGTTCTTCACGGACTTCT
EgWRKY59	ACCTCCGACACTGGAGAATA	CGCTTGGAACCGAGAAGTAG
EgWRKY60	TAGACGACGAGGAGGAAGAA	GCACTGGAAGGCTCATCTAA
EgWRKY61	GGATGAGAAGACTGATGGGAAG	CCTTCGATTGAGGACTGAGAAG
EgWRKY63	CCACTGCTCCAAGAGAAGAAA	GATGGGTTTCTGCCCGTATT
EgWRKY65	AGGTGGCTACCGGAAAGTA	CTTATCGCTGGCACTCGTATC
EgWRKY66	GGTGGCAACATATGAAGGAGAA	GTGCTGGAGGTAGTTGGTAATG
EgWRKY70	GGCTACAGTGATGTGGCTAAT	AATCTTCTCTCGGTCGCTTG
EgWRKY72	CTCTTTCGGTGACGACAATCT	CCGTCGTTCTCACCATCTTT
EgWRKY73	CTTCCTGCTTCTCGGTACTAAAG	TGTCTGGCAGCATGAGAAAT
EgWRKY81	CTACGGTTGTGAGGCAAAGA	GAGTGGTGTGATGGAGGTATG
EgWRKY84	GGCTACCGATGGAGAAAGTATG	GATCGCTCGACTCGTTTCTT
